# Supplementary material for: Development of an evidence-based complex intervention for community rehabilitation of patients with hip fracture using realist review, survey and focus groups
Source: BMJ Open. 2017 Oct 11;7(10):e014362. doi: 10.1136/bmjopen-2016-014362 (PMC5652569; doi:10.1136/bmjopen-2016-014362)
Supplement: Supplementary Appendix 1 [file bmjopen-2016-014362supp001.pdf]

Appendix 1 – List of databases searched for realist review.

- MEDLINE
- MEDLINE In-Process & Other Non-Indexed Citations
- OLDMEDLINE
- EMBASE
- Cumulative Index to Nursing and Allied Health Literature (CINAHL)
- Allied and Complimentary Medicine Database (AMED)
- British Nursing Index
- Health Management Information Consortium (HMIC)
- PsychINFO
- Cochrane Central Register of Controlled Trials (CENTRAL)
- Database of Abstracts of Reviews of Effects (DARE)
- Cochrane Database of Systematic Reviews (CDSR)
- Health Technology Assessment (HTA) Database
- NHS Economic Evaluation Database (NHS EED)
- Science Citation Index
- Social Science Citation Index (SSCI)
- Index to Scientific & Technical Proceedings (ISTP)
- Physiotherapy Evidence Database (PEDro)
- BIOSIS
- System for Information on Grey Literature In Europe (SIGLE)
- Web of Knowledge Index of Theses and Dissertations
