# Supplementary material for: Development of an evidence-based complex intervention for community rehabilitation of patients with hip fracture using realist review, survey and focus groups
Source: BMJ Open. 2017 Oct 11;7(10):e014362. doi: 10.1136/bmjopen-2016-014362 (PMC5652569; doi:10.1136/bmjopen-2016-014362)
Supplement: Author's manuscript [file bmjopen-2016-014362.draft_revisions.pdf]

BMJ Open is committed to open peer review. As part of this commitment we make the peer review history of every article we publish publicly available.

When an article is published we post the peer reviewers' comments and the authors' responses online. We also post the versions of the paper that were used during peer review. These are the versions that the peer review comments apply to.

The versions of the paper that follow are the versions that were submitted during the peer review process. They are not the versions of record or the final published versions. They should not be cited or distributed as the published version of this manuscript.

BMJ Open is an open access journal and the full, final, typeset and author-corrected version of record of the manuscript is available on our site with no access controls, subscription charges or pay-per-view fees (http://bmjopen.bmj.com).

If you have any questions on BMJ Open's open peer review process please email editorial.bmjopen@bmj.com

BMJ Open

A realist approach to complex intervention development: development of an evidence-based community rehabilitation intervention for hip fracture patients.

Journal:	*BMJ Open*
Manuscript ID	bmjopen-2016-014362
Article Type:	Research
Date Submitted by the Author:	19-Sep-2016

Complete List of Authors:	Roberts, Jessica; Bangor University, North Wales Centre for Primary Care Research<br>Din, Nafees; Bangor University, North Wales Centre for Primary Care Research<br>Williams, Michelle; Bangor University<br>Hawkes, Claire; University of Warwick Warwick Medical School, Clinical Trials Unit<br>Charles, Joanna; Bangor University, Centre for Health Economics & Medicines Evaluation<br>Hoare, Zoe; Bangor University, North Wales Organisation for Randomised Trials in Health<br>Morrison, Val; School of Psychology, Bangor University<br>Alexander, Swapna; Betsi Cadwaladr University Health Board<br>Lemmey, Andrew; Bangor University, School of Sports, Health and Exercise Science<br>Sackley, Catherine; Kings College London,<br>Logan, Phillipa; The University of Nottingham, Community Health Sciences<br>Wilkinson, Clare; Bangor University, 9. North Wales Centre for Primary Care Research<br>Rycroft-Malone, Jo; Bangor University, School of Healthcare Sciences;<br>Williams, Nefyn; Bangor University, North Wales Organisation for Randomised Trials in Health

**Primary Subject Heading**:	Rehabilitation medicine
Secondary Subject Heading:	Research methods, Health services research, Qualitative research
Keywords:	Hip < ORTHOPAEDIC & TRAUMA SURGERY, realist review, survey, focus groups, intervention development, Rehabilitation medicine < INTERNAL MEDICINE

SCHOLARONE™
Manuscripts

A realist approach to complex intervention development: development of an evidence-based community rehabilitation intervention for hip fracture patients.

Jessica L Roberts PhD[1], Nafees Ud Din MSc MFDS RCS[1], Michelle Williams BA[1], Claire A Hawkes PhD RN[4], Joanna M Charles PhD[1], Zoe Hoare PhD[1], Val Morrison PhD CPsychol[3], Swapna Alexander MD FRCP[2], Andrew Lemmey PhD[5], Catherine Sackley PhD[6], Pip Logan PhD[7], Clare Wilkinson MD FRCGP[1], Jo Rycroft-Malone PhD[1], Nefyn H Williams PhD FRCGP[1,2.]

[1]School of Healthcare Sciences, Bangor University, UK

[2]Betsi Cadwaladr University Health Board, North Wales, UK

[3]School of Psychology, Bangor University, UK

[4]Warwick Clinical Trials Unit, University of Warwick, UK

[5]School of Sports, Health and Exercise Sciences, Bangor University, UK

[6] Division of Health and Social Care Research, King's College, London, UK

[7]School of Medicine, University of Nottingham, UK

Corresponding author: Dr Nefyn H Williams, North Wales Centre for Primary Care Research, Bangor University, Gwenfro 4-8, Wrexham Technology Park, Wrexham LL13 7YP, UK.

Tel: +44 (0)1978 727430 e-mail: nefyn.williams@bangor.ac.uk

Number of text pages of the entire manuscript (including figures and tables):

Number of tables: 0 *Number of figures: 2* *Word count:* abstract 231; text 4264

For peer review only - http://bmjopen.bmj.com/site/about/guidelines.xhtml

Abstract

Objectives: To develop an evidence and theory based complex intervention for improving outcomes in elderly hip fracture patients using realist review, surveys and focus groups.

Design: Complex-intervention development for testing in a Phase II feasibility study

Setting: North Wales

Participants: Surveys of therapy managers (n=13), community and hospital-based physiotherapists (n=129) and occupational therapists (n=68) throughout the UK. Focus groups with hip fracture patients (n=13), their carers (n=4) and members of the multidisciplinary rehabilitation teams in North Wales (n=13).

Results: Three programme theories were developed from the realist review, survey and focus group findings relating to important components of rehabilitation interventions: improving patient engagement by tailoring the intervention to individual needs; reducing fear of falling and improving self-efficacy to exercise and perform activities of daily living; and co-ordination of rehabilitation delivery.

An intervention to enhance usual rehabilitation was developed to target these theory areas comprising: a physical component consisting of six additional therapy sessions; and a psychological component consisting of a workbook to enhance self-efficacy and a patient held goal-setting diary for self-monitoring.

Conclusions: A realist approach has advantages in the development of evidence-based interventions and can be used in conjunction with other established methods to contribute to the development of potentially more effective interventions. A rehabilitation intervention

For peer review only

For peer review only - http://bmjopen.bmj.com/site/about/guidelines.xhtml

was developed which has been tested to assess the feasibility of trial methods for a future definitive randomised controlled trial.

Review registration: PROSPERO 2012:CRD42012003208.

Article summary

Strengths and limitations of this study:

- The MRC framework was used to develop a complex intervention for hip fracture rehabilitation which was evidence based and theoretically underpinned.
- Programme theories were not only developed from a realist review of the literature, but also from a survey and focus groups.
- The methods used to develop this rehabilitation programme are applicable to the development of other complex interventions.
- Feasibility and acceptability of the developed intervention are reported separately, but definitive evidence of effectiveness and cost-effectiveness requires a larger randomised controlled trial.

For peer review only

Background

Proximal femoral fracture, more commonly referred to as hip fracture, is a common, major health problem in old age.[1] It is strongly associated with increased age, co morbidity, prior fragility fracture, cognitive impairment, under-nutrition, decreased bone mineral density, frailty, poor physical functioning, vision problems and weight loss.[2] Mortality is high with 25% of patients dying within the following 12 months and a further 29% do not regain their previous level of functioning.[3] The cost burden to the UK economy is approximately £2.3 billion a year.[4] The National Institute of Health and Care Excellence (NICE) management guidelines[5] recommend multidisciplinary rehabilitation, which has the potential to maximise recovery, enhance quality of life and maintain independence. However, whilst individual components of such programmes show promise, there is insufficient evidence of overall effectiveness or cost-effectiveness[6-8] and an evidence based intervention has not been published.

Rehabilitation programmes for hip fracture are complex interventions due to their multi-faceted nature and the involvement of many heterogeneous factors including individual patient circumstances and co-morbidities, healthcare professionals, rehabilitation setting and social influences.[9] The interaction of these factors in the real world and how they interplay and influence each other to determine the success and failure of such programmes is poorly understood, making it difficult to identify which specific components of rehabilitation programmes are effective and under what circumstances.[10, 11] Whilst there have been many systematic reviews of hip fracture rehabilitations these are only able to evaluate the evidence of whether an intervention works and do not allow for exploration of how and why an intervention leads to its reported outcomes. Realist reviews aim to

For peer review only

elucidate the mechanisms behind an intervention and determine 'what works, for whom, in what circumstances and why?' whilst taking into account the heterogeneous nature of such interventions and the settings in which they are delivered.[12] This involves multiple steps, which starts with extracting working theories from individual studies and developing them into 'programme theories' which describe what programmes or interventions are expected to do and how they are intended to work. These are compared and contrasted to develop intermediate programme theories, which refer to propositions of how a programme is likely to produce intended outcomes. These are then tested and refined into a final list of theories, which describe the mechanism (M) or causal force that make things happen in certain circumstances or contexts (C), such as patient characteristics or place of rehabilitation, which result in desired outcomes (O).[10]

Realist reviews provide a flexible way of exploring causal relationships thus aiding our understanding of intervention mechanisms and supporting the development of potentially more effective interventions.[13, 14]

We therefore undertook a realist review of the available evidence for hip fracture rehabilitation to develop theory on the context, mechanism and outcomes of existing rehabilitation programmes, with this forming the basis for the development of our own evidence-based intervention. The development of these theory areas was performed in conjunction with a survey of current practice by UK rehabilitation health professionals and focus groups with patients, carers and multidisciplinary rehabilitation team. Whilst the MRC framework for complex interventions provides general guidelines for intervention development and supports the use of a theoretical underpinning,[9] detailed guidance on how this framework is practically applied to intervention development is lacking. To

For peer review only - http://bmjopen.bmj.com/site/about/guidelines.xhtml

contribute to bridging this knowledge gap, this paper sets out the methodology of how the evidence base was established and utilised for intervention development, linking the findings of the review, survey and focus groups to the proposed aims of our intervention and how we expected these to facilitate our intended outcomes. The developed intervention has now been tested in a Phase II feasibility study.[15]

Study Objectives

1) To undertake a realist review to identify what is already known about the important components for a multi-disciplinary rehabilitation programme following surgical treatment for hip fracture in older people, and to understand the mechanism, context and outcome of successful interventions.
2) To assess the current provision of rehabilitation programmes following hip fracture surgery in the National Health Service throughout the UK to find out what is being provided.
3) To explore the views of patients, their carers, and health professionals in multidisciplinary rehabilitation teams on: the rehabilitation that they received or provided following surgical repair of a hip fracture; how the programme could be improved; findings from the above realist review and survey.
4) To design a rehabilitation programme from the findings of the realist review, survey and focus groups.

For peer review only - http://bmjopen.bmj.com/site/about/guidelines.xhtml

Methods

Realist review

In line with the MRC framework for complex interventions[9] the first stage in developing the rehabilitation package was to identify the evidence base and develop a theoretical understanding. As outlined, we used a realist review to determine the mechanisms behind multidisciplinary rehabilitation interventions to establish which components produced the reported outcomes for specific patient groups in which circumstances.

Developing programme theories

A scoping search of systematic reviews,[3, 7, 8, 16-50] guidelines[5, 51-55] and theoretically sound primary studies[56-61] was performed to map out the important areas and research gaps. This generated a list of questions, which could be grouped under different domains relating to: patients, health care and rehabilitation teams, rehabilitation programmes and the settings in which rehabilitation was delivered. These were formulated into statements that described how these different domains interact. These statements were subsequently refined during discussions between members of the evaluation team and with other researchers in two realist evaluation workshops.

Feedback from experts in health psychology, rehabilitation and implementation research were combined with initial survey and focus group findings and the intermediate programme theories to build the context (C), mechanisms (M) and outcome (O) configurations that provided the basis for developing the final programme theories. We developed a set of bespoke data extraction forms for each stage of theory development.

For peer review only - http://bmjopen.bmj.com/site/about/guidelines.xhtml

Literature search

The literature search strategy used by the NICE guideline review of multidisciplinary rehabilitation programmes for hip fracture[5] was adapted for this review with no search filters for study design, being intentionally inclusive so as to review different types of study to formulate and test the emerging theories. Twenty-one databases were searched from inception to Feb 2013. Citation tracking and internet search engines were used to identify additional evidence as the review progressed and as new ideas emerged. Materials were retrieved purposively to answer specific questions or test specific theories until no new themes emerged.

Screening and categorisation of references

Participants of interest were elderly adults with proximal hip fracture. The intervention of interest was multidisciplinary rehabilitation following proximal hip fracture. Outcomes of interest were mortality, pain, functional status, quality of life, health utility, health service use, costs, and patients' experiences. A working definition of multidisciplinary rehabilitation was adapted from a review of intermediate care services (Box 1).[62] Separate reviewers screened identified studies for relevance and discrepancies were resolved after discussion. Potentially relevant papers were categorised according to study type, and then according to whether they were conceptually 'rich', 'thick' or 'thin'.[63, 64] The purpose of this was to make the database manageable and to build and test theories from studies with the most relevant concepts.

For peer review only - http://bmjopen.bmj.com/site/about/guidelines.xhtml

Box 1: Working definition of multidisciplinary rehabilitation used to screen sources of evidence

Purpose	Supports re-enablement of the frail elderly following proximal hip fracture to achieve their functional potential and maintain independent living where possible
Functions	A bridge between: a) the hospital and the community;<br>b) different health care sectors and personal social care<br>Views people holistically<br>Time limited
Structure	Teams based in hospitals, the community or across both sectors
Content	Treatment and therapy (to increase strength, confidence and ability to perform activities of daily living)<br>Psychological, practical and social support<br>Support/training to develop skills and strategies for self-management
Delivery	Care delivered by a multidisciplinary team or teams

Data extraction and quality assessment

Data were extracted by one reviewer and checked for accuracy by a second. We assessed study quality using the mixed methods appraisal tool (MMAT).[65] Data from effectiveness studies were exported into structured tables to show the strength and direction of the treatment effects.

For peer review only - http://bmjopen.bmj.com/site/about/guidelines.xhtml

For peer review only

Testing the theories with quantitative and qualitative evidence

Theories were refined through an iterative process comparing individual study programme theories in turn. Data for each individual study were examined in terms of the identified programme theories and the interaction between mechanisms, context and outcomes, starting with data extracted from studies that were conceptually ‘rich’ and continuing with those that were conceptually ‘thick’. The data were then examined across the different studies to detect patterns and themes for each theory in turn, and to adjudicate between components of the final programme theories.

Survey of UK Hip Fracture Centres

Survey development

A UK wide web-based survey was conducted, targeting physiotherapists, occupational therapists and hip fracture centre therapy service managers working in the rehabilitation of patients over 65 years of age who have had surgery for proximal hip fracture. NICE guidance on hip fracture rehabilitation[5] was used as the starting point for developing the questions, which were designed to provide descriptive data of how these recommendations have been operationalised in clinical practice and service organisation. In addition, we asked whether mood, self-efficacy and fear of falling were routinely assessed, and also for examples of good practice and where improvements could be made.

Data collection

Three versions of the survey were developed for hip fracture centre managers, physiotherapists and occupational therapists. The therapist versions were further subdivided according to healthcare setting: acute hospital, community hospital or community based team.

For peer review only - http://bmjopen.bmj.com/site/about/guidelines.xhtml

The survey was open for seven weeks from 06/08/2013 to 25/09/2013. We surveyed a sample of senior managers who had a strategic role in rehabilitation services for this group of patients and aimed to achieve a 10% sample of all UK hip fracture centres. Centres in Wales, Northern Ireland and England were identified from publically available information on the National Hip Fracture Database (NHFD). Hospitals in Scotland were contacted separately and directly. We purposively sampled for geographic spread and centre size. Twenty-four centres agreed to complete the survey from around the country: 11 centres were in high (>700 operations), four were in medium (400-700 operations), and five were in low activity (<400 operations) groups. The remaining four were centres in Scotland and activity data was therefore unavailable from the NHFD. In addition to telephone contact, we advertised the survey on the NHFD web site in order to obtain data from additional centres.

As there was no register or centrally held record of physiotherapists and occupational therapists working in hip fracture rehabilitation, we advertised the survey through professional bodies and on the NHFD website. Therapy service managers completing their survey were also asked to pass the survey web link on to their therapy staff.

Data Analysis

Descriptive statistics were used to provide frequency (counts, percentages) data concerning current services and practice, where the answer format provided pre-determined response options. Where the response format was open-ended, responses were coded and categorised into themes. The integrated care pathways and physiotherapy exercise sheets returned to the team were qualitatively reviewed to provide description of commonalities and differences.

For peer review only - http://bmjopen.bmj.com/site/about/guidelines.xhtml

Focus Groups

Focus groups were completed at the three acute hospital sites across North Wales within Betsi Cadwaladr University Health Board (BCUHB). Three focus groups of members of the multidisciplinary rehabilitation teams in the community and the hospital, and four focus groups for patients and their carers were organised. Informed consent procedures were followed for recruitment, as approved by UK NHS North Wales Research Ethics Committee.

Inclusion criteria for patients

- Age 65 years or older.
- Recent proximal hip fracture.
- Surgical repair by replacement arthroplasty or internal fixation 3-12 months previously.
- Have received hip fracture rehabilitation.
- Living independently prior to hip fracture.
- Capacity to give informed consent.

Eligible participants were identified from the NHFD, through the medical and nursing staff who were responsible for maintaining the databases at each site.

Data collection

Discussions were semi-structured and run by a moderator and co-moderator using a topic guide containing open-ended questions regarding experiences, perceptions and beliefs about rehabilitation following proximal hip fracture. In the professionals' focus groups,

For peer review only - http://bmjopen.bmj.com/site/about/guidelines.xhtml

patient scenarios were also used to stimulate discussion about the sort of rehabilitation patients would be likely to receive. In the later patient focus groups, we explored initial ideas for the intervention to gain feedback. The focus group discussions were digitally recorded and fully transcribed into the speaker's original language with any portions in Welsh subsequently translated into English for analysis.

Analysis, credibility, and plausibility.

The interviews were thematically analysed using the Framework approach.[66] The initial framework used was broadly developed from the theory areas identified as important to guide the realist review and it was used to index the transcripts. Coding was completed for patient/carer and professional data separately, allowing comparison of perspectives and initial analysis. The researcher developed an initial interpretation of the data using the framework and grouped the data into themes which were reviewed by a second researcher experienced in framework analysis. A third researcher reviewed the initial framework, original transcripts and the draft analysis to make final decisions on theme structure and content. The initial and third researchers agreed the final analysis. Involving three researchers in the analysis process strengthened the plausibility and credibility of the findings as the identified themes were not generated from a single person's perspective. The indexing of the transcripts and framework charts provided an audit trail connecting the themes back to the original data.

Development of the intervention

Following initial data analysis, results from the survey and focus groups were considered by the team in the context of the initial programme theories generated from the realist review.

For peer review only - http://bmjopen.bmj.com/site/about/guidelines.xhtml

For peer review only

Further discussions confirmed final programme theories and enabled the development of individual intervention components to target these areas.

Results

The results of the three components and the development of the resultant programme theories for consideration in intervention development are summarised in Fig. 1

Developing the programme theories

Programme theory 1: Improve patient engagement by tailoring the intervention according to individual needs and preferences

Elderly proximal hip fracture patients presenting with a range of pre-fracture physical and mental functioning and a variety of co-morbidities **(C)** need a rehabilitation programme that is tailored to individual needs **(M)** in order to achieve appropriate outcomes such as improved physical functioning, greater mobility, reduced disability and independent living **(O)**.

Findings from the realist review indicated that tailoring of patient care requires a detailed assessment of patients' pre-fracture level of functioning,[67, 68] current cognitive status[69] and other comorbid conditions.[70, 71] It should also involve collaborative decision making through discussion and agreement with patients, their family and carers regarding: realistic and achievable,[71] but modifiable,[72] short-term and longer term goals of rehabilitation,[73, 74] the most appropriate setting for rehabilitation suited to patients' needs and abilities,[75-77] and any adaptation of the physical environment to facilitate day to day activities.[77, 78] In addition, the provision of enhanced support through active

For peer review only - http://bmjopen.bmj.com/site/about/guidelines.xhtml

engagement of carers and rehabilitation professionals to: motivate and facilitate the regular practice of exercises and activities of daily living,[59, 60] improve health perceptions,[79] address and adjust outcome expectations,[60, 80] and address information needs.[60, 61, 81]

In the survey, tailored rehabilitation was also identified as an important aspect of service provision. The ability to be responsive and flexible to patient need was particularly highlighted by physiotherapists. Survey findings revealed that routine clinical practice was broadly in line with current guidance, but variability existed in the provision of services, especially in the community, and that psychological mediators including self-efficacy and fear of falling were not routinely assessed using validated tools. This could leave insufficient validated information with which to tailor care to the individual. The importance of tailored care was also highlighted in focus groups, with many patients finding it hard to engage in generalised strengthening exercises, preferring individualised plans that focussed on personal goals. The first emergent theme of focus groups related to variability of care provision, which was partly because of individual tailoring of treatment, but also due to geographical variation in the availability of resources. Furthermore, co-morbidities and pre-fracture functioning determined what patients were able to do and effected their attitude to exercise, which can be taken into account through individual tailoring of care plans.

For peer review only - http://bmjopen.bmj.com/site/about/guidelines.xhtml

Programme theory 2: Reducing fear of falling and improving self-efficacy to exercise and perform activities of daily living

Proximal hip fracture results in poor physical functioning, fear of falling, low mood and lack of self-efficacy **(C)** requiring improved quality and increased amount of practice of physical exercises, activities of daily living and psychological tasks **(M)** in order to gain mastery and control to improve confidence, mobility and physical functioning **(O)**.

Enhancing the practice and quality of exercise and activities of daily living has both physical and psychological components.[60, 61] This consists of supervision and coaching by health professionals in order to improve skills and confidence to promote independent and unsupervised practice[77] with resulting increase in the duration, frequency and quality of exercises for strength, balance, gait and activities of daily living. Addressing psychological concerns is also important, particularly to improve mood.[56, 82] Motivation to practise can be improved by setting appropriate, realistic goals and developing mechanisms for monitoring and providing feedback.[82, 83]

According to the survey, more rehabilitation staff resources were needed to provide this support. Although patients' cognitive status, mood, self-efficacy and fear of falling were assessed; routine assessments using validated tools were not performed everywhere and the frequency that progress was assessed varied. The importance of psychological factors was also highlighted in focus groups where a second emergent theme was facilitators and barriers to rehabilitation, one of which was the reliance on patient's own self-motivation to seek out and access services. The level of patient engagement in the rehabilitation programme also depended upon its perceived relevance to their day-to-day activities and in the absence of this, amount of practice was likely to decline. A third focus group theme was

For peer review only - http://bmjopen.bmj.com/site/about/guidelines.xhtml

the psychosocial effects of the fracture, fear of falling in particular, which reduced confidence and increased the reliance on walking aids. This fear not only affected engagement in the rehabilitation programme but also with wider social interactions, leading to feelings of isolation.

Programme theory 3: Co-ordination of services and sectors delivering the rehabilitation

The diversity of services provided by different disciplines, across sectors from a variety of funders **(C)** requires a co-ordinated provision of the multidisciplinary rehabilitation programme **(M)** in order to deliver appropriate physical, functional and psychological interventions to patients in a timely manner **(O)**.

This coordination of multidisciplinary care from the acute hospital into the community, requires improved communication between rehabilitation professionals and careful discharge planning. Patients valued the help and support they received from health care teams during their recovery and regarded this as the single most important factor in their recovery, so the provision of consistent and reliable care is vital.

Respondents in the survey from both acute and community hospital settings mostly reported that routine clinical practice was following the latest NICE (2011)[5] and SIGN (2009)[51] guidance. Multidisciplinary teams working with common goals across settings were a strength, but there was variability in service provision, especially what was available in the community. Liaison between the acute hospital and the community could be improved, as could communication with patients and carers.

The fourth focus group theme was a need for more information for patients and their carers about what to expect following the hip fracture and how to access all of the available

For peer review only - http://bmjopen.bmj.com/site/about/guidelines.xhtml

resources. This was because the complexity in programme provision and often poor communication between different sectors, meant that rehabilitation was neither smooth nor seamless, and because of this lack of consistency, patients felt unsupported in their recovery. Patients and their carers required reassurance from qualified professionals about which activities were safe to perform in order to overcome these barriers, highlighting the role of the therapist as a mediator to improve their own self-efficacy.

Designing a rehabilitation intervention

Considering these findings, we developed a programme comprising both physical and psychological components. The physical component consisted of additional rehabilitation sessions tailored to individual need, following discharge home. These increased the opportunity for practice and professional support, with the aim of improving overall mobility, independence and functional outcomes. The psychological component consisted of a patient-held information workbook, developed using an existing stroke rehabilitation workbook[84, 85] as an exemplar, and a goal-setting diary. These aimed to improve patient engagement in the rehabilitation programme by giving patients a sense of ownership of their own recovery by:

- Enhancing self-efficacy through goal setting for motivation and increased participation in rehabilitation trajectory
- Self-monitoring and feedback on goals
- Verbal encouragement and support by professionals
- Providing information on what to expect from recovery
- Increasing confidence through reassurance and encouraging patients to seek advice.

For peer review only - http://bmjopen.bmj.com/site/about/guidelines.xhtml

The additional sessions were also an opportunity for patients to obtain reassurance and guidance from a qualified healthcare professional. Similarly, the outcome of the psychological components aimed to increase confidence and self-efficacy that would affect patient's ability and willingness to perform exercises, thus improving their physical outcomes.

The individual intervention components were designed to target the programme theories which were identified as important components of successful hip fracture rehabilitation programmes (Fig. 2). A detailed logic model of the intervention activities, their proposed long and short-term goals and how these target different components of the International Classification of Functioning framework has previously been published,[15] along with how the intervention addresses specific areas of existing NICE guidance for hip fracture rehabilitation.

Discussion

There were three programme theories from the realist review: improving patient engagement by tailoring the intervention according to individual needs and preferences; reducing fear of falling and improving self-efficacy to exercise and perform activities of daily living; and co-ordination of services and sectors delivering the rehabilitation. These were reflected in the survey data highlighting that whilst routine clinical practice was broadly in line with current guidance, there was variability in the provision of services, especially in the community, and that important psychological mediators such as self-efficacy and fear of falling were not routinely assessed using validated tools. They also agreed with the four focus group themes of: variation in rehabilitation care provided; the need for more

For peer review only - http://bmjopen.bmj.com/site/about/guidelines.xhtml

For peer review only

information; facilitators and barriers to rehabilitation; and the psychosocial impact of hip fracture. These findings informed the development of a community-based rehabilitation intervention consisting of a psychological component delivered using a workbook and a patient-held goal setting diary and a physical component comprising additional rehabilitation sessions.

This was the first realist review of rehabilitation following hip fracture and the first UK wide survey aiming to describe rehabilitation for patients following hip fracture across acute and community settings since the introduction of NICE recommendations for rehabilitation in 2011.[5] As a realist review rather than a systematic review was performed we did not attempt to summarise all evidence and judge whether rehabilitation programmes were effective, but rather sought to build an explanatory account of mechanisms behind rehabilitation and to establish which components were considered to be effective and in which circumstances. Whilst a good range of respondents were sampled in the survey, it was not possible to sample settings, therapists and community service managers proportionately, which may impact on how representative findings are of the whole UK. Similarly, focus groups findings relate specifically to the location we recruited from as this was the proposed setting for the delivery of the enhanced rehabilitation programme. We had also hoped to purposively sample patients with different levels of disability and who had received different types of rehabilitation, however, it was not possible to identify these criteria from electronic medical records. Participants had a range of ages and experiences across the groups, though we were unable to sample those who were living independently prior to hip fracture, but who now lived in residential or nursing care, and those with cognitive impairment.

For peer review only - http://bmjopen.bmj.com/site/about/guidelines.xhtml

Other studies have acknowledged the benefits of using realist review in intervention development,[86] but such methods have not previously been used in hip fracture rehabilitation research. A qualitative study exploring mobility levels pre- and post-fracture also reported fear of falling, lack of confidence and reliance on others as having an impact on patient experiences of rehabilitation.[87] The patient need for information from healthcare professionals and the importance of this in successful rehabilitation has also been previously identified.[88] A qualitative study of physiotherapist perceptions of rehabilitation also found that there is tailoring of care to patient's individual needs, based on their own goals and level of support available.[89] A study into the challenges of team working in rehabilitation of hip fracture patients also demonstrated that there were breakdowns in communication within multidisciplinary teams and issues relating to the organisation of resources and services, which led to variation in patient care.[90]

Implications for future research

This study demonstrates the potential benefits of using a realist approach to complex intervention development and how a realist review can be used in conjunction with other established methods to provide an evidence base for a hip fracture rehabilitation intervention. This approach may be beneficial for developing complex interventions in other clinical areas and can be used to provide theories of how specific intervention components will facilitate their intended outcomes. The next phase in the MRC framework for evaluating complex interventions[9] was to test the feasibility of methods for a future trial of the developed intervention by testing it's acceptability in a phase II feasibility study.[15] This has been completed and the intervention deemed feasible and acceptable to patients, carers and healthcare professionals delivering the intervention (in press, BMJ Open).

For peer review only - http://bmjopen.bmj.com/site/about/guidelines.xhtml

Review registration

Williams N, Din N, Rycroft-Malone J, Wilkinson C, Edwards R, Charles J. Fracture in the Elderly Multidisciplinary Rehabilitation (FEMuR): developing a multidisciplinary rehabilitation package following hip fracture. PROSPERO 2012:CRD42012003208. Available from http://www.crd.york.ac.uk/PROSPERO/display_record.asp?ID=CRD42012003208. 2012[15]

Declarations

Ethics, consent and permissions

Ethical approval was granted for the study by UK NHS North Wales Research Ethics Committee – West. Ref 12/WA/0355. All participants gave informed consent to participate in accordance with the procedures approved by the committee.

Consent for publication

Individual data has been anonymised, and consent was obtained for use in publication.

Availability of data

There is no additional unpublished data available.

Conflict of interest statement

None declared.

Funding

For peer review only - http://bmjopen.bmj.com/site/about/guidelines.xhtml

This research was funded by the Health Technology Assessment (HTA) programme of the National Institute of Health Care Research (NIHR). (HTA reference 11/33/03). The views and opinions expressed therein are those of the authors and do not necessarily reflect those of the HTA programme, NIHR, the National Health Service, or the Department of Health.

Author contributions

NHW – CI responsible for study design, conduct and analysis, led intervention development, led writing of manuscript and is guarantor; JLR – conducted focus group analysis and contributed to survey analysis, led writing of manuscript, development of intervention materials; NUD – conducted realist review, contributed to writing manuscript; MW – conducted survey analysis, contributed to writing of manuscript; CH – trial management, input to study and survey design, oversight of intervention development, conducted focus groups and survey, initial focus group analysis; JC – assisted with realist review; ZH – input to study design, design of survey and initial survey analysis; VM – Co-I responsible for study design, provided health psychology expertise and methodological oversight; SA – Co-I, consultant orthogeriatrician, provided orthogeriatric expertise and input on intervention design; AL – Co-I responsible for study design, provided methodological oversight; CS – Co-I contributing to methodology and study design, provided physiotherapy and rehabilitation expertise and input on intervention design; PL - Co-I contributing to methodology and study design, provided physiotherapy and rehabilitation expertise and input on intervention design; CW – Co-I contributing to study design and methodology; JRM – Co-I providing realist review expertise and methodological input. All authors were involved in writing and reviewing of the manuscript and decisions on final content.

Acknowledgements

For peer review only - http://bmjopen.bmj.com/site/about/guidelines.xhtml

For peer review only

We would like to thank the patients, carers and staff who took part in the focus groups and survey, especially Glenys Mutter who maintains the Hip Fracture Database at Ysbyty Glan Clwyd and assisted us with identifying potential focus group participants.

We would also like to thank:

Dr Julia Hiscock for assistance with focus groups and consultation on qualitative analysis; Aaron Pritchard for consultation on qualitative analysis; Barbara France for running the electronic literature searches and study document retrieval; Annie Hendry for help in extracting the data from the included studies; Dr Richa Sinha for helping during reference screening and conceptual categorisation of potentially relevant studies for the realist review and John Spalding/ Bangor University library staff for helping in electronically inaccessible document retrieval through inter library loans.

For peer review only

References

1. Johansen A, Wakeman R, Boulton C, et al. National hip fracture database. National report 2013. London: Royal College of Physicians, 2013.
2. National Osteoporosis Foundation. Osteoporosis: Review of the evidence for prevention, diagnosis and treatment and cost-effectiveness analysis. Executive summary. *Osteoporosis Int*. 1998;4(S3-6).
3. Bertram M, Norman R, Kemp L, et al. Review of the long-term disability associated with hip fractures. *Injury Prev*. 2011;17(6):365-70.
4. National Osteoporosis Society. Key facts and figures. Accessed september 2014. Available at http://www.Nos.Org.Uk/page.Aspx?Pid=328.
5. National clinical guideline centre. Hip fracture: The management of hip fracture in adults | guidance and guidelines | nice. National institute of health and care excellence 2011. Accessed 2014. Available at http://www.Nice.Org.Uk/guidance/cg124.
6. Handoll HH, Cameron ID, Mak JC, et al. Multidisciplinary rehabilitation for older people with hip fractures. *Cohrane Database Syst Rev*. 2009(4).
7. Handoll HH, Sherrington C, Mak JC. Interventions for improving mobility after hip fracture surgery in adults. *Cohrane Database Syst Rev*. 2011(3).
8. Crotty M, Unroe K, Cameron ID, et al. Rehabilitation interventions for improving physical and psychosocial functioning after hip fracture in older people. *Cohrane Database Syst Rev*. 2010(1).
9. Council MR. Developing and evaluating complex interventions: New guidance. 2008.
10. Wong G, Westhorp G, Pawson R, et al. Realist synthesis: Ramses training materials. 2013; Available from: http://www.ramesesproject.org/media/Realist_reviews_training_materials.pdf.
11. Wong G, Greenhalgh T, Westhorp G, et al. Development of methodological guidance, publication standards and training materials for realist and meta-narrative reviews: The rameses (realist and meta-narrative evidence syntheses – evolving standards) project. Southampton (UK). *NIHR Journals Library*;Sep 2014.
12. Pawson R, Greenhalgh T, Harvey G, et al. Realist review - a new method of systematic review designed for complex policy interventions. *J Health Serv Res Policy*. 2005;10.
13. Wong G, Greenhalgh T, Westhorp G, et al. Realist methods in medical education research: What are they and what can they contribute? *Med Educ*. 2012;46.
14. Rycroft-Malone J, Mccormack B, Hutchinson AM, et al. Realist synthesis: Illustrating the method for implementation research. *Implement Sci*. 2012;7.
15. Williams NH, Hawkes C, Din NU, et al. Fracture in the elderly multidisciplinary rehabilitation (femur): Study protocol for a phase ii randomised feasibility study of a multidisciplinary rehabilitation package following hip fracture [isrctn22464643]. *Pilot and Feasibility Studies*. 2015;1(1):1-22.
16. Lafferty G. Community-based alternatives to hospital rehabilitation services: A review of the evidence and suggestions for approaching future evaluations. *Rev Clin Gerontol*. 1996;6(02):183-94.
17. Handoll HHG, Sherrington C. Mobilisation strategies after hip fracture surgery in adults. *Cohrane Database Syst Rev*. 2007(1):CD001704.
18. Ward D, Et Al. Care home versus hospital and own home environments for rehabilitation of older people. *Cohrane Database Syst Rev,*. 2008;DOI: 10.1002/14651858.CD003164.pub2.
19. Cameron ID, Handoll HH, Finnegan TP, et al. Co-ordinated multidisciplinary approaches for inpatient rehabilitation of older patients with proximal femoral fractures. *Cohrane Database Syst Rev*. 2001(3):CD000106.
20. Auais M, Eilayyan O, Mayo NE. Extended exercise rehabilitation after hip fracture improves patients' physical function: A systematic review and meta-analysis. *Physical Therapy*. 2012.
21. Beaupre LA, Allyson Jones C, Duncan Saunders L, et al. Best practices for elderly hip fracture patients. *J Gen Intern Med*. 2005;20(11):1019-25.

For peer review only - http://bmjopen.bmj.com/site/about/guidelines.xhtml

22. Cameron ID. Coordinated multidisciplinary rehabilitation after hip fracture. *Disabil Rehabil*. 2005;27(18/19):1081-90.

23. Chudyk AM, Jutai JW, Petrella RJ, et al. Systematic review of hip fracture rehabilitation practices in the elderly. *Arch Phys Med Rehabil*. 2009;90(2):246-62.

24. Feehan LM, Beck CA, Harris SR, et al. Exercise prescription after fragility fracture in older adults: A scoping review. *Osteoporosis Int*. 2011;22(5):1289-322.

25. Halbert J, Crotty M, Whitehead C, et al. Multi-disciplinary rehabilitation after hip fracture is associated with improved outcome: A systematic review. *Journal of Rehabilitation Medicine (Stiftelsen Rehabiliteringsinformation)*. 2007;39(7):507-12.

26. Leigheb F, Vanhaecht K, Sermeus W, et al. The effect of care pathways for hip fractures: A systematic review. *Calcif Tissue Int*. 2012;91(1):1-14.

27. Mehta SP, Roy J-S. Systematic review of home physiotherapy after hip fracture surgery. *Journal of Rehabilitation Medicine (Stiftelsen Rehabiliteringsinformation)*. 2011;43(6):477-80.

28. Moffa-Trotter ME, Anemaet WK. Cost effectiveness of home rehabilitation: A literature review. *Top Geriatr Rehabil*. 1999;14(4):1-33.

29. Nigwekar SU, Rajda J, Navaneethan SD. Hospitalist care and length of stay in patients with hip fracture: A systematic review. *Archives of Internal Medicine*. 2008;168(9):1010-1.

30. Sherrington C. The effects of exercise on physical ability following fall-related hip fracture. PhD Thesis, University of New South Wales (Australia). 2001.

31. Smith TO, Hedges C, Macnair R, et al. Early rehabilitation following less invasive surgical stabilisation plate fixation for distal femoral fractures. *Physiotherapy*. 2009;95(2):61-75.

32. Toussant EM, Kohia M. A critical review of literature regarding the effectiveness of physical therapy management of hip fracture in elderly persons. *J Gerontol A Biol Sci Med*. 2005;60(10):1285-91.

33. Yamamoto N. Rehabilitation and mobilization in hip fracture patients. *Clin Calcium*. 2010;20(9):1402-6.

34. Allen J, Koziak A, Buddingh S, et al. Rehabilitation in patients with dementia following hip fracture: A systematic review. *Physiotherapy Canada*. 2012;64(2):190-201.

35. Bachmann S, Finger C, Huss A, et al. Inpatient rehabilitation specifically designed for geriatric patients: Systematic review and meta-analysis of randomised controlled trials. *BMJ*. 2010;340:c1718.

36. Cameron I, Crotty M, Currie C, et al. Geriatric rehabilitation following fractures in older people: A systematic review. *Health Technology Assessment*. 2000;4(2):105.

37. Cameron ID. Models of rehabilitation -- commonalities of interventions that work and of those that do not. *Disabil Rehabil*. 2010;32(12):1051-8.

38. Chong CP, Savige J, Lim WK. Orthopaedic-geriatric models of care and their effectiveness. *Australasian Journal on Ageing*. 2009;28(4):171-6.

39. Drenth JC, J.S.M. H. Assessment of physical fitness using upper extremity tests in elderly patients during the non-weight bearing stage after hip fracture: A systematic review *[Dutch] Nederlands Tijdschrift Voor Fysiotherapie*. 2012;122(3):104-10.

40. Egan M JS, Byrne K, Wells J, Stolee P. Factors associated with a second hip fracture: A systematic review. *Clin Rehabil*. 2008;22:272-82.

41. Egol KA, Koval KJ, Zuckerman JD. Functional recovery following hip fracture in the elderly. *J Orthop Trauma*. 1997;11(8):594-9.

42. Green J, Et Al. Burden and medical needs in older patients with hip fractures and muscle atrophy or weakness. *Osteoporosis Int*. 2012;23:S93-S4.

43. Hu F, Jiang C, Shen J, et al. Preoperative predictors for mortality following hip fracture surgery: A systematic review and meta-analysis. *Injury*. 2012;43(6):676-85.

44. Jelicic M, Kempen GIJ, Van Eijk LM. Do psychosocial factors affect recovery from hip fracture in the elderly? A review of the literature. *Journal of Rehabilitation Sciences*. 1996;9(3):77-81.

45. Mauffrey C. The management of subcapital fractures in the elderly population. *Eur J Orthop Surg Traumatol*. 2009;20(5):359-64.

For peer review only

46. Muir SW, Yohannes AM. The impact of cognitive impairment on rehabilitation outcomes in elderly patients admitted with a femoral neck fracture: A systematic review. *J Geriatr Phys Ther*. 2009;32(1):24-32.
47. Robinson KM. Efficacy of home care rehabilitation interventions. *Ann Longterm Care*. 2000;8(9):68-71.
48. Romeo R, Knapp M, Banerjee S, et al. Treatment and prevention of depression after surgery for hip fracture in older people: Cost-effectiveness analysis. *J Affect Disord*. 2011;128(3):211-9.
49. Visschedijk J, Achterberg W, Van Balen R, et al. Fear of falling after hip fracture: A systematic review of measurement instruments, prevalence, interventions, and related factors. *Journal of the American Geriatrics Society*. 2010;58(9):1739-48.
50. Wang X, Emery LJ. Cognitive status after hip replacement. *Physical & Occupational Therapy in Geriatrics*. 2002;21(1):51-64.
51. Scottish intercollegiate guidelines network (sign) management of hip fracture in older people: A national clinical guideline. *Edinburgh, SIGN*. 2010.
52. Hip Fractures. October 2014. Agency for Healthcare Research and Quality R, Md. Http://Www.Ahrq.Gov/Research/Findings/Evidence-Based-Reports/Hipfractp.Html.
53. Health quality ontario; ministry of health and long-term care. Quality-based procedures: Clinical handbook for hip fracture. Toronto, on: Health quality ontario; 2013 may. 97 p. Available from: Http://www.Hqontario.Ca/evidence/publications-and-ohtac-rec.
54. Minimum standards for the management of hip fracture in the older person. Agency for clinical innovation, new south wales, austrailia. Available at http://www.Aci.Health.Nsw.Gov.Au/__data/assets/pdf_file/0004/222727/minimum-standards-for-the-management-of-hip-fractures.Pdf.
55. New zealand guidelines group (2003). Acute management and immediate rehabilitation after hip fracture amongst people ages 65 years and over. Wellington, new zealand. Available at http://www.Moh.Govt.Nz/notebook/nbbooks.Nsf/0/bf485cca409f38c5cc256dce0070f7b5/$file/hip_fracture_management.Pdf.
56. Resnick B, D'adamo C, Shardell M, et al. Adherence to an exercise intervention among older women post hip fracture. *Journal of clinical sport psychology*. 2008;2(1):41-56.
57. Resnick B, Daly MP. The effect of cognitive status on outcomes following rehabilitation. *Fam Med*. 1997;29(6):400-5.
58. Resnick B, Magaziner J, Orwig D, et al. Evaluating the components of the exercise plus program: Rationale, theory and implementation. *Health Educ Res*. 2002;17(5):648-58.
59. Resnick B, Orwig D, Hawkes W, et al. The relationship between psychosocial state and exercise behavior of older women 2 months after hip fracture. *Rehabil Nurs*. 2007;32(4):139-49.
60. Resnick B, Orwig D, Wehren L, et al. The exercise plus program for older women post hip fracture: Participant perspectives. *The Gerontologist*. 2005;45(4):539-44.
61. Resnick B, Orwig D, Yu-Yahiro J, et al. Testing the effectiveness of the exercise plus program in older women post-hip fracture. *Ann Behav Med*. 2007;34(1):67-76.
62. Pearson M HH, Cooper C, Shepperd S, Pawson R, Anderson R. Intermediate care: A realist review and conceptual framework. 2013.
63. Ritzer G. Meta-theorizing in sociology. Lexington, MA: Lexington Books; 1991.
64. Roen K, Arai L, Roberts H, et al. Extending systematic reviews to include evidence on implementation: Methodological work on a review of community-based initiatives to prevent injuries. *Soc Sci Med*. 2006;63(4):1060-71.
65. Pluye P, Robert E, Cargo M EA. Proposal: A mixed methods appraisal tool for systematic mixed studies reviews. 2011.
66. Ritchie J and Spencer L. Qualitative data analysis for applied policy research. In: Analysing qualitative data. London: Routledge; 1994. p. 173–94.

For peer review only - http://bmjopen.bmj.com/site/about/guidelines.xhtml

67. Bellelli G, Buccino G, Bernardini B, et al. Action observation treatment improves recovery of postsurgical orthopedic patients: Evidence for a top-down effect? *Arch Phys Med Rehabil*. 2010;91(10):1489-94.

68. Saltvedt I, Prestmo A, Einarsen E, et al. Development and delivery of patient treatment in the trondheim hip fracture trial. A new geriatric in-hospital pathway for elderly patients with hip fracture. *BMC Res Notes*. 2012;5:355.

69. Rolland Y, Pillard F, Lauwers-Cances V, et al. Rehabilitation outcome of elderly patients with hip fracture and cognitive impairment. *Disabil Rehabil*. 2004;26(7):425-31.

70. Stenvall M, Olofsson B, Nyberg L, et al. Improved performance in activities of daily living and mobility after a multidisciplinary postoperative rehabilitation in older people with femoral neck fracture: A randomized controlled trial with 1-year follow-up. *Journal of Rehabilitation Medicine (Stiftelsen Rehabiliteringsinformation)*. 2007;39(3):232-8.

71. Young Y, Resnick B. Don't worry, be positive: Improving functional recovery 1 year after hip fracture. *Rehabil Nurs*. 2009;34(3):110-7.

72. Jackson J, Schkade J. Occupational adaptation model versus biomechanical-rehabilitation model in the treatment of patients with hip fractures. *Am J Occup Ther*. 2001;55(5):531-7.

73. Godfrey M, Townsend J. Older people in transition from illness to health: Trajectories of recovery. *Qual Health Res*. 2008;18(7):939-51.

74. Manthorpe J, Cornes M. Intermediate care: Older people's involvement and experiences. *Journal of Integrated Care*. 2004;12(6):43-8.

75. Crotty M, Whitehead C, Gray S, et al. Early discharge and home rehabilitation after hip fracture achieves functional improvements: A randomized controlled trial. *Clin Rehabil*. 2002;16(4):406-13.

76. Sirkka M, Bränholm I. Consequences of a hip fracture in activity performance and life satisfaction in an elderly swedish clientele. *Scand J Occup Ther*. 2003;10(1):34-9.

77. Ziden L, Wenestam C, Hansson-Scherman M. A life-breaking event: Early experiences of the consequences of a hip fracture for elderly people. *Clin Rehabil*. 2008;22(9):801-11.

78. Bauerle D, Specht-Leible N, Voss E. Hip fracture - changes in need of help and care - cluster analysis - formal and informal support. [german] veranderungen des hilfe- und pflegebedarfs nach huftnahen frakturen im hoheren lebensalter. *Z Gerontol Geriatr*. 2004;37(5):351-3.

79. Huang T, Acton G. Ways to maintain independence among taiwanese elderly adults with hip fractures: A qualitative study. *Geriatric Nursing*. 2009;30(1):28-35.

80. Lieberman D, Friger M. Inpatient rehabilitation outcome after hip fracture surgery in elderly patients: A prospective cohort study of 956 patients. *Arch Phys Med Rehabil*. 2006;87(2):167-71.

81. Mcmillan L, Booth J, Currie K, et al. A grounded theory of taking control after fall-induced hip fracture. *Disabil Rehabil*. 2012;34(26):2234-41.

82. Resnick B, Orwig D, D'adamo C, et al. Factors that influence exercise activity among women post hip fracture participating in the exercise plus program. *Clin Interv Aging*. 2007;2(3):413-27. Epub 2007/11/30.

83. Bandura A. Self-efficacy: The exercise of control. New York, NY, US: W H Freeman/Times Books/ Henry Holt & Co; 1997. ix, 604 p.

84. Morrison VL, Johnston M, Macwalter RS, et al. Improving emotional outcomes following acute stroke: A preliminary evaluation of a work-book based intervention. *Scott Med J*. 1998;43(2):52-3.

85. Johnston M, Bonetti D, Joice S, et al. Recovery from disability after stroke as a target for a behavioural intervention: Results of a randomized controlled trial. *Disabil Rehabil*. 2007;29(14):1117-27.

86. Pearson M, Brand SL, Quinn C, et al. Using realist review to inform intervention development: Methodological illustration and conceptual platform for collaborative care in offender mental health. *Implement Sci*. 2015;10:134. Epub 2015/09/30.

For peer review only - http://bmjopen.bmj.com/site/about/guidelines.xhtml

87. Taylor NF, Barelli, C and Harding, K.E. Community ambulation before and after hip fracture: A qualitative analysis. *Disabil Rehabil*. 2010;32(15):1281-90.

88. Mcmillan L, Booth J, Currie K, et al. 'Balancing risk' after fall-induced hip fracture: The older person's need for information. *Int J Older People Nurs*. 2014;9(4):249-57.

89. Taylor NF, Harding, K.E., Dowling, J., Harrison, G. Discharge planning for patients receiving rehabilitation after hip fracture: A qualitative analysis of physiotherapists' perceptions. *Disability and Rehabilitation*. 2010;32(6):492-9.

90. Long AF, Kneafsey R, Ryan J. Rehabilitation practice: Challenges to effective team working. *Int J Nurs Stud*. 2003;40(6):663-73.

Fig 1. Development of programme theories for informing the content of the enhanced rehabilitation intervention.

Fig. 2. Proposed outcomes of individual intervention components target specific programme theories.

For peer review only

For peer review only - http://bmjopen.bmj.com/site/about/guidelines.xhtml

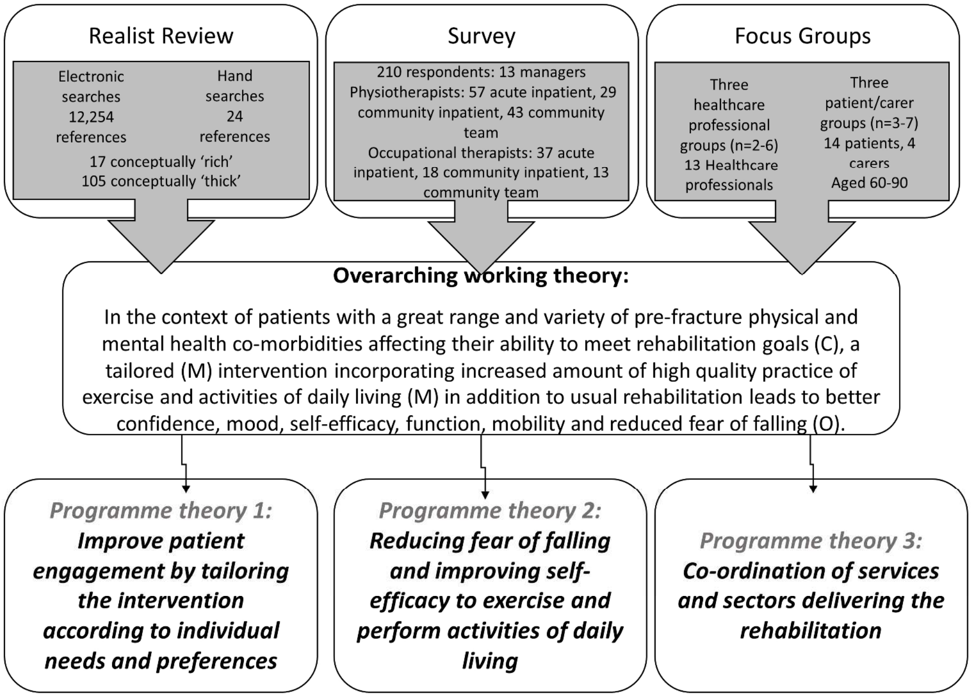

Fig 1. Development of programme theories for informing the content of the enhanced rehabilitation intervention.

254x190mm (300 x 300 DPI)

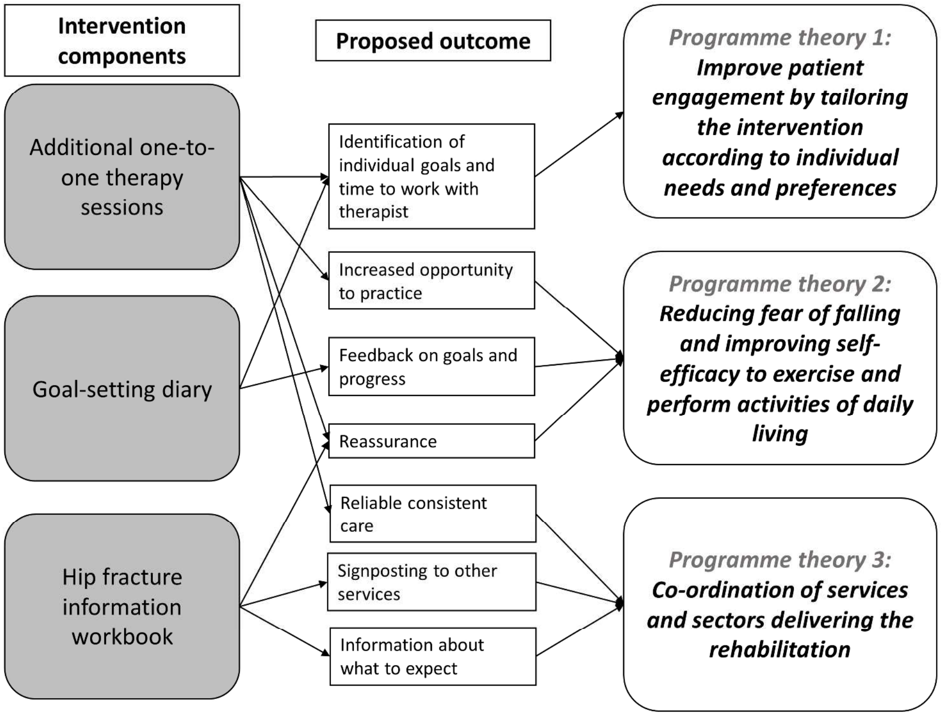

Fig. 2. Proposed outcomes of individual intervention components target specific programme theories.

254x190mm (300 x 300 DPI)

BMJ Open

Development of an evidence-based complex intervention for community rehabilitation of hip fracture patients using realist review, survey and focus groups

Journal:	*BMJ Open*
Manuscript ID	bmjopen-2016-014362.R1
Article Type:	Research
Date Submitted by the Author:	20-Jun-2017
Complete List of Authors:	Roberts, Jessica; Bangor University, North Wales Centre for Primary Care Research<br>Din, Nafees; Bangor University, North Wales Centre for Primary Care Research<br>Williams, Michelle; Bangor University<br>Hawkes, Claire; University of Warwick Warwick Medical School, Clinical Trials Unit<br>Charles, Joanna; Bangor University, Centre for Health Economics & Medicines Evaluation<br>Hoare, Zoe; Bangor University, North Wales Organisation for Randomised Trials in Health<br>Morrison, Val; School of Psychology, Bangor University<br>Alexander, Swapna; Betsi Cadwaladr University Health Board<br>Lemmey, Andrew; Bangor University, School of Sports, Health and Exercise Science<br>Sackley, Catherine; Kings College London,<br>Logan, Phillipa; The University of Nottingham, Community Health Sciences<br>Wilkinson, Clare; Bangor University, 9. North Wales Centre for Primary Care Research<br>Rycroft-Malone, Jo; Bangor University, School of Healthcare Sciences;<br>Williams, Nefyn; Bangor University, North Wales Organisation for Randomised Trials in Health
**Primary Subject Heading**:	Rehabilitation medicine
Secondary Subject Heading:	Research methods, Health services research, Qualitative research
Keywords:	Hip < ORTHOPAEDIC & TRAUMA SURGERY, realist review, survey, focus groups, intervention development, Rehabilitation medicine < INTERNAL MEDICINE

SCHOLARONE™
Manuscripts

Development of an evidence-based complex intervention for community rehabilitation of hip fracture patients using realist review, survey and focus groups

Jessica L Roberts PhD[1], Nafees Ud Din MSc MFDS RCS[1], Michelle Williams BA[1], Claire A Hawkes PhD RN[4], Joanna M Charles PhD[1], Zoe Hoare PhD[1], Val Morrison PhD CPsychol[3], Swapna Alexander MD FRCP[2], Andrew Lemmey PhD[5], Catherine Sackley PhD[6], Phillipa Logan PhD[7], Clare Wilkinson MD FRCGP[1], Jo Rycroft-Malone PhD[1], Nefyn H Williams PhD FRCGP[1,2.]

[1]School of Healthcare Sciences, Bangor University, UK

[2]Betsi Cadwaladr University Health Board, North Wales, UK

[3]School of Psychology, Bangor University, UK

[4]Warwick Clinical Trials Unit, University of Warwick, UK

[5]School of Sports, Health and Exercise Sciences, Bangor University, UK

[6] Division of Health and Social Care Research, King's College, London, UK

[7]School of Medicine, University of Nottingham, UK

Corresponding author: Dr Jessica Roberts, North Wales Centre for Primary Care Research, Bangor University, Cambrian 2, Wrexham Technology Park, Wrexham LL13 7YP, UK.

Tel: +44 (0)1248 383516 e-mail: j.l.roberts@bangor.ac.uk

For peer review only

Abstract

Objectives: To develop an evidence and theory based complex intervention for improving outcomes in elderly hip fracture patients.

Design: Complex-intervention development (Medical Research Council [MRC] framework phase I) using realist literature review, surveys and focus groups of patients and rehabilitation teams.

Setting: North Wales

Participants: Surveys of therapy managers (n=13), community and hospital-based physiotherapists (n=129) and occupational therapists (n=68) throughout the UK. Focus groups with hip fracture patients (n=13), their carers (n=4) and members of the multidisciplinary rehabilitation teams in North Wales (n=13).

Results: The realist review provided understanding of how rehabilitation interventions work in the real world context and three programme theories were developed: improving patient engagement by tailoring the intervention to individual needs; reducing fear of falling and improving self-efficacy to exercise and perform activities of daily living; and co-ordination of rehabilitation delivery. The survey provided context about usual rehabilitation practice; focus groups provided data on the experience, acceptability and feasibility of rehabilitation interventions.

An intervention to enhance usual rehabilitation was developed to target these theory areas comprising: a physical component consisting of six additional therapy sessions; and a psychological component consisting of a workbook to enhance self-efficacy and a patient held goal-setting diary for self-monitoring.

For peer review only - http://bmjopen.bmj.com/site/about/guidelines.xhtml

For peer review only

Conclusions: A realist approach may have advantages in the development of evidence-based interventions and can be used in conjunction with other established methods to contribute to the development of potentially more effective interventions. A rehabilitation intervention was developed which can be tested in a future randomised controlled trial (MRC framework phases II and III).

Review registration: PROSPERO 2012:CRD42012003208.

Article summary

Strengths and limitations of this study:

- A complex intervention for hip fracture rehabilitation was developed, which was evidence based and theoretically underpinned (Medical Research Council [MRC] framework phase I).
- Programme theories were developed from a realist review of the literature. A survey added context, and focus groups provided data on the experience, acceptability and feasibility of rehabilitation interventions.
- The methods used to develop this rehabilitation programme may be applicable to the development of other complex interventions.
- The feasibility and acceptability of the developed intervention are reported separately, but evidence of effectiveness and cost-effectiveness requires testing in a randomised controlled trial (MRC framework phases II and III).

For peer review only

For peer review only - http://bmjopen.bmj.com/site/about/guidelines.xhtml

Background

Proximal femoral fracture, more commonly referred to as hip fracture, is a common, major health problem in the elderly [1]. It is associated with prior fragility fracture, and other co-morbidities such as: cognitive impairment, under-nutrition, decreased bone mineral density, frailty, poor physical functioning, vision problems and weight loss [2]. Mortality is high with 14-58% of patients dying within 12 months [3, 4] and up to 53% do not regain their previous level of functioning [5, 6]. Proximal femoral fractures cost the UK economy approximately £2.3 billion a year [7]. Management guidelines from the National Institute of Health and Care Excellence (NICE) [8] recommend multidisciplinary rehabilitation, which has the potential to maximise recovery, enhance quality of life and maintain independence. However, systematic reviews conclude that there is insufficient evidence of overall effectiveness or cost-effectiveness, but individual components of such programmes may show promise [9-11].

Rehabilitation programmes for hip fracture are complex interventions due to their multi-faceted nature and the involvement of many heterogeneous factors including individual patient circumstances and co-morbidities, healthcare professionals, rehabilitation setting and social influences [12]. The interaction of these factors in the real world and how they interplay and influence each other to determine the success and failure of such programmes is poorly understood, making it difficult to identify which specific components of rehabilitation programmes are effective and under what circumstances [13, 14]. Whilst there have been many systematic reviews of hip fracture rehabilitations [5, 10, 11], these are only able to evaluate the evidence of whether an intervention works and do not allow for exploration of how and why an intervention leads to its reported outcomes. Realist

For peer review only - http://bmjopen.bmj.com/site/about/guidelines.xhtml

reviews aim to elucidate the mechanisms behind an intervention and determine ‘what works, for whom, in what circumstances, and why?’ whilst taking into account the heterogeneous nature of such interventions and the settings in which they are delivered [15]. This involves multiple steps, which starts with extracting working theories from individual studies and developing them into ‘programme theories’ which describe what programmes or interventions are expected to do and how they are intended to work. These are compared and contrasted to develop intermediate programme theories, which refer to propositions of how a programme is likely to produce intended outcomes. These are then tested and refined into a final list of theories, which describe the mechanism (M) or causal force that makes things happen in certain circumstances or contexts (C), such as patient characteristics or place of rehabilitation, which result in desired outcomes (O) [13].

Realist reviews provide a flexible way of exploring causal relationships, thus aiding our understanding of intervention mechanisms and supporting the development of potentially more effective interventions [16, 17].

We therefore undertook a realist review of the available evidence for hip fracture rehabilitation to develop theory on the context, mechanism and outcomes of existing rehabilitation programmes, with this forming the basis for the development of our own evidence-based intervention for subsequent testing in a feasibility study [18]. The development of these theory areas was performed in conjunction with a survey of current practice by UK rehabilitation health professionals and focus groups with patients, carers and multidisciplinary rehabilitation team. Whilst the MRC framework for complex interventions provides general guidelines for intervention development and supports the use of theoretical underpinning [12], detailed guidance on how this framework is practically

For peer review only - http://bmjopen.bmj.com/site/about/guidelines.xhtml

applied to intervention development is lacking. To contribute to bridging this knowledge gap, this paper sets out the methodology of how the evidence base was established and utilised for intervention development, linking the findings of the review, survey and focus groups to the proposed aims of our intervention and how we expected these to facilitate our intended outcomes.

Methods

The development of the community-based rehabilitation package was informed by three complementary work packages. A coherent theoretical basis for the intervention was developed from a realist literature review. Initial findings from the review were used to develop the questions for a survey. A survey of current services described usual practice, and was an additional source of relevant theories that contributed to the realist review. The initial findings from the review were also used to develop topic guides for the focus groups and the initial framework for their analysis. Focus groups provided data on the experience, acceptability and feasibility of rehabilitation interventions.

Realist review

A realist review was used to identify the evidence base and develop a theoretical understanding. A scoping search of systematic reviews [5, 10, 11, 19-53], guidelines [8, 54-58] and theoretically rich primary studies [59-64] was performed to map out the important areas and research gaps (NUD, NHW). This generated a list of questions, which could be

For peer review only - http://bmjopen.bmj.com/site/about/guidelines.xhtml

grouped under different domains relating to: patients, healthcare and rehabilitation teams, rehabilitation programmes, and the settings in which rehabilitation was delivered.

The list of questions were formulated into statements that described how these different domains interact. These statements were subsequently refined during discussions between members of the research team (JR-M, NUD, NHW, JMC) and with other researchers from the School of Healthcare Sciences, Bangor University, in two realist review workshops.

Feedback from experts in health psychology, rehabilitation and implementation research were combined with initial survey and focus group findings into candidate programme theories. These intermediate working theories were used as the basis for bespoke data extraction forms.

Literature search

The literature search strategy used by the NICE guideline review of multidisciplinary rehabilitation programmes for hip fracture [8] was adapted for this review, with no search filters for study design; this intentional inclusivity enabled review of different types of study, which, in turn, facilitated formulation and examination of the emerging theories. Twenty one databases were searched from inception to Feb 2013 (Appendix 1). Citation tracking and internet search engines were used to identify additional evidence as the review progressed and as new ideas emerged. Materials were retrieved purposively to answer specific questions or test specific theories until no new themes emerged.

Screening and categorisation of references

Participants of interest were elderly adults with proximal hip fracture. The intervention of interest was multidisciplinary rehabilitation following proximal hip fracture. Outcomes of

For peer review only - http://bmjopen.bmj.com/site/about/guidelines.xhtml

interest were mortality, pain, functional status, quality of life, health utility, health service use, costs, and patients' experiences. A working definition of multidisciplinary rehabilitation was adapted from a review of intermediate care services [65] (Box 1). Separate reviewers screened identified studies for relevance and discrepancies were resolved after discussion (NUD, NHW, JC). Potentially relevant papers were categorised according to study type, and then according to whether they were conceptually 'rich', 'thick' or 'thin' [66, 67] (NUD, NHW). We started by extracting data from all of the 'rich' studies and sampled data from the conceptually 'thick' studies until saturation was reached. The purpose of this was to make the database manageable and to build and examine theories from studies with the most relevant concepts.

For peer review only

For peer review only - http://bmjopen.bmj.com/site/about/guidelines.xhtml

Box 1: Working definition of multidisciplinary rehabilitation used to screen sources of evidence

Purpose	Supports re-enablement of the frail elderly (over 65 years old) following proximal hip fracture to achieve their functional potential and maintain independent living where possible
Functions	A bridge between: a) the hospital and the community;<br>b) different healthcare sectors and personal social care<br>Views people holistically<br>Time limited (up to one year following fracture)
Structure	Teams based in hospitals, the community or across both sectors
Content	Treatment and therapy (to increase strength, confidence and ability to perform activities of daily living)<br>Psychological, practical and social support<br>Support/training to develop skills and strategies for self-management
Delivery	Care delivered by a multidisciplinary team or teams

Data extraction and quality assessment

Data were extracted by one reviewer (NUD) and checked for accuracy by a second (NHW). We assessed study quality using the mixed methods appraisal tool (MMAT) [68]. Data from effectiveness studies were exported into structured tables to show the strength and direction of the treatment effects.

For peer review only - http://bmjopen.bmj.com/site/about/guidelines.xhtml

Testing the theories with quantitative and qualitative evidence

Theories were refined through an iterative process comparing individual study programme theories in turn. Data for each individual study were examined in terms of the identified programme theories and the interaction between mechanisms, context and outcomes, starting with data extracted from studies that were conceptually 'rich' and continuing with those that were conceptually 'thick'. The data were then examined across the different studies to detect patterns and themes. A second set of refined date extraction forms were used to test each theory in turn, and adjudicate between components of the final programme theories.

Survey of UK Hip Fracture Centres

Survey development

A UK wide web-based survey was conducted, targeting physiotherapists, occupational therapists and hip fracture centre therapy service managers working in the rehabilitation of patients over 65 years of age who have had surgery for proximal hip fracture. NICE guidance on hip fracture rehabilitation [5] was used with initial findings from the scoping review as the starting point for developing the questions. The questionnaires were piloted on members of staff across one health board in Wales, and minor amendments were made.

Data collection

Three versions of the survey were developed for hip fracture centre managers, physiotherapists and occupational therapists. The managers' survey focused on the organisation of services, whilst the therapists' questionnaires focussed on aspects of clinical practice such as session content, frequency and location, and how assessments were

For peer review only - http://bmjopen.bmj.com/site/about/guidelines.xhtml

conducted. The therapists' versions were further subdivided according to healthcare setting: acute hospital, community hospital or community based team.

The survey was open for seven weeks from 06/08/2013 to 25/09/2013. We surveyed a sample of senior managers who had a strategic role in rehabilitation services for this group of patients and aimed to achieve a 10% sample of all UK centres performing hip fracture surgery. Centres in Wales, Northern Ireland and England were identified from publically available information on the National Hip Fracture Database (NHFD). Hospitals in Scotland were contacted separately and directly. We purposively sampled for geographic spread and centre size, contacting centres by telephone and through advertising on the NHFD. Twenty four centres from across the UK agreed to take part.

Data Analysis

Descriptive statistics were used to provide frequency (counts, percentages) data concerning current services and practice, where the answer format provided pre-determined response options (ZH). Where the response format was open-ended, responses were coded and categorised into themes (MW). The integrated care pathways and physiotherapy exercise sheets returned to the team were qualitatively reviewed to provide description of commonalities and differences (MW).

Focus Groups

Focus groups were completed at the three acute hospital sites across North Wales within Betsi Cadwaladr University Health Board (BCUHB). Three focus groups of members of the multidisciplinary rehabilitation teams in the community and the hospital, and four focus

For peer review only - http://bmjopen.bmj.com/site/about/guidelines.xhtml

groups for patients and their carers were organised. Informed consent procedures were followed for recruitment, as approved by UK NHS North Wales Research Ethics Committee.

People who were over 65 years, were receiving rehabilitation following surgical repair of a proximal hip fracture within the last 3-12 month, were living independently prior to fracture, and were able to provide informed consent were eligible to take part.

Eligible participants were identified from the NHFD, through the medical and nursing staff who were responsible for maintaining the database at each site.

Data collection

Discussions were semi-structured and run by a moderator (CH) and co-moderator (MW, NHW, JLR or NUD) using a topic guide containing open-ended questions regarding experiences, perceptions and beliefs about rehabilitation following proximal hip fracture. In the professionals' focus groups, patient scenarios were also used to stimulate discussion about the sort of rehabilitation patients would be likely to receive. In the later patient focus groups, we explored initial ideas for the intervention to gain feedback. The focus group discussions were digitally recorded and fully transcribed into the speaker's original language, with any portions in Welsh subsequently translated into English for analysis. The interviews were thematically analysed using the Framework approach [69]. The initial framework used was broadly developed from the theory areas identified as important to guide the realist review and it was used to index the transcripts. The researcher developed an initial interpretation of the data using the framework, and grouped the data into themes which were reviewed by a second researcher experienced in framework analysis. A third researcher reviewed the initial framework, original transcripts and the draft analysis to

For peer review only - http://bmjopen.bmj.com/site/about/guidelines.xhtml

make final decisions on theme structure and content. The initial and third researchers agreed the final analysis.

Development of the intervention

The final programme theories and the results from the survey and the initial focus groups were discussed by all of the research team in order to identify the important components of the rehabilitation intervention. The intervention components were discussed and refined in the final focus groups.

For peer review only

For peer review only - http://bmjopen.bmj.com/site/about/guidelines.xhtml

Results

The literature review, survey results and qualitative data were used to develop the following overarching working theory;

> "In the context of patients with a great range and variety of pre-fracture physical and mental health co-morbidities affecting their ability to meet rehabilitation goals (C), a tailored (M) intervention incorporating increased quality and amount of practice of exercise and activities of daily living (M) in addition to usual rehabilitation leads to better confidence, mood, self-efficacy, function, mobility and reduced fear of falling (O)."

This was then broken down into three component programme theories described below.

(Figure 1).

Programme theory 1: Improve patient engagement by tailoring the intervention according to individual needs and preferences

Elderly proximal hip fracture patients presenting with a range of pre-fracture physical and mental functioning and a variety of co-morbidities **(C)** need a rehabilitation programme that is tailored to individual needs **(M)** in order to achieve appropriate outcomes such as improved physical functioning, greater mobility, reduced disability and independent living **(O)**.

Findings from the realist review indicated that tailoring of patient care requires a detailed assessment of patients' pre-fracture level of functioning [70, 71], current cognitive status [72] and other co-morbid conditions [73, 74]. It should also involve collaborative decision making through discussion and agreement with patients, their family and carers regarding:

For peer review only - http://bmjopen.bmj.com/site/about/guidelines.xhtml

realistic and achievable [74], but modifiable [75], short-term and longer term goals of rehabilitation [76, 77], the most appropriate setting for rehabilitation suited to patients' needs and abilities [78-80], and any adaptation of the physical environment to facilitate day to day activities [80, 81]. In addition, the provision of enhanced support through active engagement of carers and rehabilitation professionals to motivate and facilitate the regular practice of exercises and activities of daily living [62, 63], improve health perceptions [82], address and adjust outcome expectations [63, 83], and address information needs [63, 64, 84].

In the survey, tailored rehabilitation was also identified as an important aspect of service provision. Respondents from all categories reported that the frequency of rehabilitation visits received were influenced by individual patient need (Fig 2.). Survey findings revealed that routine clinical practice was broadly in line with current guidance, but variability existed in the provision of services, especially in the community. Variation was reported in the frequency of rehabilitation visits following hip fracture (Fig. 2), with some services performing multiple visits a day and others visiting patients less than once a week. There was also variation in the types of activities included. For example, whilst the majority of occupational therapists (over 95%) included prescribing equipment and practising activities of daily living in their usual activities, less than 50% included anxiety management and developing self-awareness. The importance of tailored care was also highlighted in focus groups (Table 1), with many patients finding it hard to engage in strengthening exercises if they were not part of an individualised plan that focussed on personal goals. The first emergent theme from the focus groups related to the variability of care provision, which was partly because of individual tailoring of treatment, but also geographical variation in the

For peer review only - http://bmjopen.bmj.com/site/about/guidelines.xhtml

availability of resources. Furthermore, co-morbidities and pre-fracture functioning determined what patients were able to do and affected their attitude to exercise, which could be taken into account through individual tailoring of care plans.

Table 1. Focus group themes and supporting quotes from patients, carers and healthcare professionals

Theme	Supporting quotes
**Variation of rehabilitation care provision**	*"It depends completely on the patient you can't just say well this is what is going to happen to every patient, they vary so much… there is different avenues depending on what they present"* R4, community hospital physiotherapist, FG1121.<br><br>*"It depends on what, what procedure she [the surgeon] has done to fix the fractured NOF [neck of femur] as to what level of interventions we do"* R2, occupational therapist, FG1321.<br><br>*"You are dealing with very angry relatives who were under the presumption that because they are under our service, that they will automatically get care and they won't, not unless there is a need"* R1, clinical specialist physiotherapist, FG1321.
**Facilitators and barriers to rehabilitation**	*"There's a limit to what you can do at home, I got to the stage where I needed equipment… the first time I went to the gym and saw the physio there, I thought yes...It hurt, it was painful, but at least I felt I'm sure I'm going to get somewhere, and it has it's been brilliant."* F2, female patient, FG1111<br><br>*"We refer a lot [to falls group], as long as they can get transport"* (R, FG1221).<br><br>*"Seeing the physio, it's a mixture of more exercises and going through it but also it's the ability just to have someone to talk through things like what to do with the pain"* M1, male patient, FG1111

For peer review only - http://bmjopen.bmj.com/site/about/guidelines.xhtml

**Psychosocial impact of hip fracture**	*"Couple of women recently and have taken ages, whereas initially talking to them they are women you know sort of retired but really active, do loads, but then they have fallen and really I think it's more, you know the shock of the falling over and not being able to do things it does take them quite a long time to get over it."* R3, acute hospital occupational therapist, FG1121.<br><br>*"You think you are going to fall all the time, erm… so it is just practice I think, just keep doing it, keep doing little bits and erm…I had the reassurance from the physiotherapist who said 'no, by next summer you will be doing exactly what you were doing last summer.'"* R1, female patient, FG1212.<br><br>*"It's to do with personal care as well, and to raise confidence as well, that's a lot to do with it because people who have had the falls, it's their confidence really that's taken a big knock"* R3, reablement team, FG1221.
**Need for information**	*"I didn't know what to do I didn't know whether to sit, and rest or try to exercise or what nobody told me anything… people don't explain… tell you so that you can understand. You just, left to ponder it over for yourself"* R3, female patient, FG1211.<br><br>*"There was a whole series of questions I had that had come up over the previous three weeks and I think the ability to go and talk to someone, with different experience and knowledge was very important for me now"* M1, male patient, FG1111<br><br>*"Care is good, communication is rubbish"* I3, male carer, FG1311.

For peer review only - http://bmjopen.bmj.com/site/about/guidelines.xhtml

Programme theory 2: Reducing fear of falling and improving self-efficacy to exercise and perform activities of daily living

Proximal hip fracture results in poor physical functioning, fear of falling, low mood and lack of self-efficacy **(C)** requiring improved quality and increased amount of practice of physical exercises, activities of daily living and psychological tasks **(M)** in order to gain mastery and control to improve confidence, mobility and physical functioning **(O)**.

Enhancing the practice and quality of exercise and activities of daily living has both physical and psychological components [63, 64]. This consists of supervision and coaching by health professionals in order to improve skills and confidence to promote independent and unsupervised practice [80], with resulting increases in the duration, frequency and quality of exercises for improving strength, balance, gait and activities of daily living. Addressing psychological concerns is also important, particularly to improve mood [59, 85]. Motivation to practise can be improved by setting appropriate, realistic goals and developing mechanisms for monitoring and providing feedback [85, 86].

According to the survey, more rehabilitation staff resources were needed to provide this support. Although patients' cognitive status, mood, self-efficacy and fear of falling were assessed; routine assessments using validated tools were not performed everywhere and the frequency that progress was assessed varied (Table 2). The importance of psychological factors was also highlighted in focus groups where a second emergent theme was facilitators and barriers to rehabilitation, one of which was the reliance on patient's own self-motivation to seek out and access services. The level of patient engagement in the rehabilitation programme depended upon its perceived relevance to their day-to-day activities, and in the absence of this the amount of practice was likely to decline. A third

For peer review only - http://bmjopen.bmj.com/site/about/guidelines.xhtml

Table 2. Validated measures reported to be used by physiotherapists and occupational therapists in acute and community settings.

	Acute hospital	Community Hospital	Community Team
**Assessment**	**Tools used**	**Tools used**	**Tools used**
**Cognitive status**	Abbreviated mental test score (AMTS).<br>Mini mental state examination (MMSE).<br>Rapid assessment test for delirium and cognitive impairment (4AT)<br>Six Item Cognitive Impairment Test (6CIT).<br>Montreal Cognitive Assessment (MoCA).<br>Addenbrooke's Cognitive Examination (ACE-R).	Six Item Cognitive Impairment Test (6CIT).<br>Montreal Cognitive Assessment (MoCA).<br>Abbreviated mental test score (AMTS).MMSE<br>Middlesex Elderly Assessment of Mental State (MEAMS).	Six Item Cognitive Impairment Test (6CIT).<br>Addenbrooke's Cognitive Examination (ACE-R).<br>Mini mental state examination (MMSE).<br>Middlesex Elderly Assessment of Mental State (MEAMS).<br>Montreal Cognitive Assessment (MoCA).<br>COG test<br>Rowland Universal Dementia Assessment Scale (RUDAS).
**Mood**	Hospital Anxiety and Depression Scale (HADS).<br>Geriatric Depression Scale (GDS).<br>Unified assessment proforma.<br>Montreal Cognitive Assessment (MoCA).	Geriatric Depression Scale (GDS).Hospital Anxiety and Depression Scale (HADS).<br>The 12-Item Short Form Health Survey (SF-12).<br>Canadian Occupational Performance Measure (COPM).	Six Item Cognitive Impairment Test (6CIT).<br>Geriatric Depression Scale (GDS).<br>Depression Scale (HADS).<br>Therapy Outcome Measure (TOM).
**Self-efficacy**	Unified assessment proforma.<br>10m walk.	The Falls Efficacy Scale (FES)<br>Visual Analogue Scale (VAS).<br>Canadian Occupational Performance Measure (COPM).	Euro Qol (EQ-5D).<br>Therapy Outcome Measure (TOM).
**Fear of falling**	Visual Analogue Scale (VAS).<br>Berg Balance Scale.<br>Elderly mobility scale.<br>Oxford hip scale.<br>Tinetti Assessment Tool.	Falls Risk Assessment Tool (FRAT).<br>Visual Analogue Scale (VAS).<br>The Falls Efficacy Scale (FES).<br>The Falls Efficacy Scale International (FES-I).<br>Tinetti Assessment Tool.<br>Canadian Occupational Performance Measure (COPM).	Falls Risk Assessment Tool (FRAT).Visual Analogue Scale (VAS).
**Health utility**		Euro Qol (EQ-5D).	Euro Qol (EQ-5D).

For peer review only - http://bmjopen.bmj.com/site/about/guidelines.xhtml

focus group theme was the psychosocial effects of the fracture, fear of falling in particular, which reduced confidence and increased the reliance on walking aids. This fear not only affected engagement in the rehabilitation programme but also impacted on wider social interactions, leading to feelings of isolation.

Programme theory 3: Co-ordination of services and sectors delivering the rehabilitation

The diversity of services provided by different disciplines, across sectors from a variety of funders **(C)** requires a co-ordinated provision of the multidisciplinary rehabilitation programme **(M)** in order to deliver appropriate physical, functional and psychological interventions to patients in a timely manner **(O)**.

The co-ordination of multidisciplinary care from the acute hospital into the community, required good communication between rehabilitation professionals and careful discharge planning. Patients valued the help and support they received from healthcare teams during their recovery and regarded this as the single most important factor in their recovery, so the provision of consistent and reliable care was vital.

Most respondents in the survey from both acute and community hospital settings reported that routine clinical practice was following the latest NICE (2011) [8] and SIGN (2009) [54] guidance. Multidisciplinary teams working with common goals across settings were a strength, but there was variability in service provision, especially with regard to what was available in the community. Liaison between the acute hospital and the community could be improved, as could communication with patients and carers.

The fourth focus group theme was a need for more information for patients and their carers about what to expect following the hip fracture and how to access all of the available

For peer review only - http://bmjopen.bmj.com/site/about/guidelines.xhtml

resources. The complexity in programme provision and the often poor communication between different sectors, meant that rehabilitation was neither smooth nor seamless, and because of this lack of consistency, patients felt unsupported in their recovery. Patients and their carers required reassurance from qualified professionals about which activities were safe to perform in order to overcome these barriers, highlighting the role of the therapist as a mediator to improve their self-efficacy.

Designing a rehabilitation intervention

Considering these findings the rehabilitation intervention needed to:

- identify individual goals with help from a therapist;
- enhance self-efficacy;
- increase the opportunity to practise prescribed exercises and activities of daily living;
- support the self-monitoring of progress towards identified goals;
- give encouragement and support from professionals;
- provide information on what to expect during rehabilitation;
- provide reliable and consistent care;
- sign post to other available services.

In order to address these, we developed a rehabilitation programme comprising both physical and psychological components (Fig. 3). The physical component consisted of additional rehabilitation sessions, tailored to individual need, following discharge home. The psychological component consisted of a patient-held information workbook, developed using an existing stroke rehabilitation workbook [87, 88] as an exemplar, and

For peer review only - http://bmjopen.bmj.com/site/about/guidelines.xhtml

a goal-setting diary. These aimed to improve patient engagement in the rehabilitation programme by giving patients a sense of ownership of their own recovery.

The additional sessions were also an opportunity for patients to obtain reassurance and guidance from a qualified healthcare professional. Similarly, the outcome of the psychological components aimed to increase confidence and self-efficacy that would affect patient's ability and willingness to perform exercises, thus improving their physical outcomes.

A detailed logic model of the intervention activities, their proposed long and short-term goals and how these target different components of the International Classification of Functioning framework has previously been published [89], along with how the intervention addresses specific areas of existing NICE guidance for hip fracture rehabilitation.

Discussion

There were three programme theories from the realist review: improving patient engagement by tailoring the intervention according to individual needs and preferences; reducing fear of falling and improving self-efficacy to exercise and perform activities of daily living; and co-ordination of services and sectors delivering the rehabilitation. These were reflected in the survey data highlighting that whilst routine clinical practice was broadly in line with current guidance, there was variability in the provision of services, especially in the community, and that important psychological mediators such as self-efficacy and fear of falling were not routinely assessed using validated tools. They also agreed with the four focus group themes of: variation in rehabilitation care provided; the need for more information; facilitators and barriers to rehabilitation; and the psychosocial impact of hip

For peer review only - http://bmjopen.bmj.com/site/about/guidelines.xhtml

fracture. These findings informed the development of a community-based rehabilitation intervention consisting of a psychological component delivered using a workbook and a patient-held goal setting diary and a physical component comprising additional rehabilitation sessions.

Other studies have acknowledged the benefits of using realist review in intervention development [90], but such methods have not previously been used in hip fracture rehabilitation research. This paper adds to the understanding of how a realist review can be used in conjunction with other methods to develop complex interventions which link individual intervention components with underlying programme theories.

The findings from the individual work packages are supported by existing literature. A qualitative study of physiotherapists' perceptions of rehabilitation also showed that tailoring of care to patient's individual needs, based on their own goals and level of support available, was an important component of successful rehabilitation [91]. A previous qualitative study exploring mobility levels pre- and post-fracture also reported that fear of falling, lack of confidence and reliance on others had an impact on patients' experiences of rehabilitation [92]. This study highlighted the need to include psychological components in rehabilitation interventions, supporting our findings about the importance of improving self-efficacy and confidence in hip fracture patients. Our finding concerning patients' need for information from healthcare professionals and its importance in successful rehabilitation has also been previously identified [93]. A study into the challenges of team working in the rehabilitation of hip fracture patients found that there were breakdowns in communication within multidisciplinary teams and issues relating to the organisation of resources and

For peer review only - http://bmjopen.bmj.com/site/about/guidelines.xhtml

services, which led to variation in patient care [94]. Our intervention aims to address this by co-ordinating care through the means of a patient-held goal-setting diary.

This was the first realist review of rehabilitation following hip fracture and the first UK wide survey aiming to describe rehabilitation for patients following hip fracture across acute and community settings since the introduction of NICE recommendations for rehabilitation in 2011 [5]. As a realist review rather than a systematic review was performed we did not attempt to summarise all of the evidence and judge whether rehabilitation programmes were effective, but rather sought to build an explanatory account of mechanisms behind rehabilitation and to establish which components were considered to be effective and in which circumstances. Whilst a good range of respondents were sampled in the survey, it was not possible to sample settings, therapists and community service managers proportionately, which may impact on how representative findings are of the whole UK. Similarly, focus groups findings relate specifically to the location we recruited from as this was the proposed setting for the delivery of the enhanced rehabilitation programme. We had also hoped to purposively sample patients with different levels of disability who had received different types of rehabilitation, however it was not possible to identify these criteria from electronic medical records. Participants had a range of ages and experiences across the groups, though we were unable to sample those who were living independently prior to hip fracture, but who now lived in residential or nursing care, and those with cognitive impairment.

Implications for future research

This study demonstrated the potential benefits of using a realist approach to complex intervention development and how a realist review could be used in conjunction with other

For peer review only

established methods to provide an evidence base for a hip fracture rehabilitation intervention. This approach may be beneficial for developing complex interventions in other clinical areas and can be used to provide theories of how specific intervention components will facilitate their intended outcomes. The next phase in the MRC framework for evaluating complex interventions [12] was to test the feasibility of methods for a future trial of the developed intervention by testing its acceptability in a phase II feasibility study [89, 95].

Review registration

Williams N, Din N, Rycroft-Malone J, Wilkinson C, Edwards R, Charles J. Fracture in the Elderly Multidisciplinary Rehabilitation (FEMuR): developing a multidisciplinary rehabilitation package following hip fracture. PROSPERO 2012:CRD42012003208. Available from http://www.crd.york.ac.uk/PROSPERO/display_record.asp?ID=CRD42012003208. 2012.

Declarations

Ethics, consent and permissions

Ethical approval was granted for the study by UK NHS North Wales Research Ethics Committee – West. Ref 12/WA/0355. All participants gave informed consent to participate in accordance with the procedures approved by the committee.

Consent for publication

Individual data has been anonymised, and consent was obtained for use in publication.

Availability of data

For peer review only - http://bmjopen.bmj.com/site/about/guidelines.xhtml

The datasets generated and analysed during the current study are available from the corresponding author on reasonable request.

Conflict of interest statement

None declared.

Funding

This research was funded by the Health Technology Assessment (HTA) programme of the National Institute for Health Research (NIHR). (HTA reference 11/33/03). The views and opinions expressed therein are those of the authors and do not necessarily reflect those of the HTA programme, NIHR, the National Health Service, or the Department of Health.

Author contributions

NHW – CI responsible for study design, conduct and analysis, led intervention development, led writing of manuscript and is guarantor; JLR – conducted focus group analysis and contributed to survey analysis, led writing of manuscript, development of intervention materials; NUD – conducted realist review, contributed to writing manuscript; MW – conducted survey analysis, contributed to writing of manuscript; CH – trial management, input to study and survey design, oversight of intervention development, conducted focus groups and survey, initial focus group analysis; JC – assisted with realist review; ZH – input to study design, design of survey and initial survey analysis; VM – Co-I responsible for study design, provided health psychology expertise and methodological oversight; SA – Co-I, consultant orthogeriatrician, provided orthogeriatric expertise and input on intervention design; AL – Co-I responsible for study design, provided methodological oversight; CS – Co-I contributing to methodology and study design, provided physiotherapy and rehabilitation

For peer review only - http://bmjopen.bmj.com/site/about/guidelines.xhtml

For peer review only

expertise and input on intervention design; PL - Co-I contributing to methodology and study design, provided physiotherapy and rehabilitation expertise and input on intervention design; CW – Co-I contributing to study design and methodology; JRM – Co-I providing realist review expertise and methodological input. All authors were involved in writing and reviewing of the manuscript and decisions on final content.

Acknowledgements

We would like to thank the patients, carers and staff who took part in the focus groups and survey, especially Glenys Mutter who maintains the Hip Fracture Database at Ysbyty Glan Clwyd and assisted us with identifying potential focus group participants.

We would also like to thank:

Dr Julia Hiscock for assistance with focus groups and consultation on qualitative analysis; Aaron Pritchard for consultation on qualitative analysis; Barbara France for running the electronic literature searches and study document retrieval; Annie Hendry for help in extracting the data from the included studies; Dr Richa Sinha for helping during reference screening and conceptual categorisation of potentially relevant studies for the realist review; Patricia Masterson Allgar and Professor Christopher Burton who were participants in the realist review workshops; John Spalding/ Bangor University library staff for helping in electronically inaccessible document retrieval through inter library loans.

For peer review only - http://bmjopen.bmj.com/site/about/guidelines.xhtml

References

1. Johansen A, et al., *National Hip Fracture Database. National report 2013*, C.E.a.E. Unit, Editor 2013, Royal College of Physicians: London.
2. National Osteoporosis Foundation *Osteoporosis: review of the evidence for prevention, diagnosis and treatment and cost-effectiveness analysis. Executive summary.* Osteoporosis International, 1998. **4**(S3-6).
3. Schnell, S., et al., *The 1-year mortality of patients treated in a hip fracture program for elders.* Geriatr Orthop Surg Rehabil, 2010. **1**(1): p. 6-14.
4. Roche, J.J., et al., *Effect of comorbidities and postoperative complications on mortality after hip fracture in elderly people: prospective observational cohort study.* BMJ, 2005. **331**(7529): p. 1374.
5. Bertram, M., et al., *Review of the long-term disability associated with hip fractures.* Injury Prevention, 2011. **17**(6): p. 365-370.
6. Harvey, N., E. Dennison, and C. Cooper, *Osteoporosis: impact on health and economics.* Nat Rev Rheumatol, 2010. **6**(2): p. 99-105.
7. *National Osteoporosis Society. Key facts and figures. Accessed September 2014. Available at http://www.nos.org.uk/page.aspx?pid=328*.
8. National Clinical Guideline Centre. *Hip fracture: the management of hip fracture in adults | Guidance and guidelines | NICE. National Institute of Health and Care Excellence 2011. Accessed 2014. Available at http://www.nice.org.uk/guidance/cg124*.
9. Handoll, H.H., et al., *Multidisciplinary rehabilitation for older people with hip fractures.* Cochrane Database of Systematic Reviews, 2009(4).
10. Handoll, H.H., C. Sherrington, and J.C. Mak, *Interventions for improving mobility after hip fracture surgery in adults.* Cochrane Database of Systematic Reviews, 2011(3).
11. Crotty, M., et al., *Rehabilitation interventions for improving physical and psychosocial functioning after hip fracture in older people.* Cochrane Database of Systematic Reviews, 2010(1).
12. Medical Research Council, *Developing and evaluating complex interventions: new guidance*, 2008.
13. Wong, G., et al. *Realist Synthesis: RAMESES Training Materials*. 2013; Available from: http://www.ramesesproject.org/media/Realist_reviews_training_materials.pdf.
14. Wong, G., et al., *Development of methodological guidance, publication standards and training materials for realist and meta-narrative reviews: the RAMESES (Realist And Meta-narrative Evidence Syntheses – Evolving Standards) project.*, H.S.a.D. Research., Editor 2014, NIHR Journals Library.: Southampton (UK).
15. Pawson, R., et al., *Realist review - a new method of systematic review designed for complex policy interventions.* J Health Serv Res Policy, 2005. **10**.
16. Wong, G., et al., *Realist methods in medical education research: what are they and what can they contribute?* Med Educ, 2012. **46**.
17. Rycroft-Malone, J., et al., *Realist synthesis: illustrating the method for implementation research.* Implement Sci, 2012. **7**.
18. Williams, N.H., et al., *Developing a multidisciplinary rehabilitation package following hip fracture and testing in a randomised feasibility study: Fracture in the Elderly Multidisciplinary Rehabilitation (FEMuR).* Health Technology Assessment, 2017. **21**.
19. Lafferty, G., *Community-based alternatives to hospital rehabilitation services: a review of the evidence and suggestions for approaching future evaluations.* Reviews in Clinical Gerontology, 1996. **6**(02): p. 183-194.
20. Handoll, H.H.G. and C. Sherrington, *Mobilisation strategies after hip fracture surgery in adults.* Cochrane Database of Systematic Reviews, 2007(1): p. CD001704.

For peer review only

21. Ward, D., et al., *Care home versus hospital and own home environments for rehabilitation of older people.* Cochrane Database of Systematic Reviews,, 2008. **DOI: 10.1002/14651858.CD003164.pub2.**
22. Cameron, I.D., et al., *Co-ordinated multidisciplinary approaches for inpatient rehabilitation of older patients with proximal femoral fractures.* Cochrane Database of Systematic Reviews, 2001(3): p. CD000106.
23. Auais, M., O. Eilayyan, and N.E. Mayo, *Extended Exercise Rehabilitation After Hip Fracture Improves Patients' Physical Function: A Systematic Review and Meta-Analysis.* Physical Therapy, 2012.
24. Beaupre, L.A., et al., *Best Practices for Elderly Hip Fracture Patients.* Journal of General Internal Medicine, 2005. **20**(11): p. 1019-1025.
25. Cameron, I.D., *Coordinated multidisciplinary rehabilitation after hip fracture.* Disability & Rehabilitation, 2005. **27**(18/19): p. 1081-1090.
26. Chudyk, A.M., et al., *Systematic review of hip fracture rehabilitation practices in the elderly.* Archives of Physical Medicine & Rehabilitation, 2009. **90**(2): p. 246-262.
27. Feehan, L.M., et al., *Exercise prescription after fragility fracture in older adults: a scoping review.* Osteoporosis International, 2011. **22**(5): p. 1289-322.
28. Halbert, J., et al., *Multi-disciplinary rehabilitation after hip fracture is associated with improved outcome: a systematic review.* Journal of Rehabilitation Medicine (Stiftelsen Rehabiliteringsinformation), 2007. **39**(7): p. 507-512.
29. Leigheb, F., et al., *The Effect of Care Pathways for Hip Fractures: A Systematic Review.* Calcified Tissue International, 2012. **91**(1): p. 1-14.
30. Mehta, S.P. and J.-S. Roy, *Systematic review of home physiotherapy after hip fracture surgery.* Journal of Rehabilitation Medicine (Stiftelsen Rehabiliteringsinformation), 2011. **43**(6): p. 477-480.
31. Moffa-Trotter, M.E. and W.K. Anemaet, *Cost Effectiveness of Home Rehabilitation: A Literature Review.* Topics in Geriatric Rehabilitation, 1999. **14**(4): p. 1-33.
32. Nigwekar, S.U., J. Rajda, and S.D. Navaneethan, *Hospitalist care and length of stay in patients with hip fracture: A systematic review.* Archives of Internal Medicine, 2008. **168**(9): p. 1010-1011.
33. Sherrington, C., *The effects of exercise on physical ability following fall-related hip fracture.* University of New South Wales (Australia), 2001: p. p. pages unknown.
34. Smith, T.O., et al., *Early rehabilitation following less invasive surgical stabilisation plate fixation for distal femoral fractures.* Physiotherapy, 2009. **95**(2): p. 61-75.
35. Toussant, E.M. and M. Kohia, *A Critical Review of Literature Regarding the Effectiveness of Physical Therapy Management of Hip Fracture in Elderly Persons.* The Journals of Gerontology Series A: Biological Sciences and Medical Sciences, 2005. **60**(10): p. 1285-1291.
36. Yamamoto, N., *Rehabilitation and mobilization in hip fracture patients.* Clinical Calcium, 2010. **20**(9): p. 1402-6.
37. Allen, J., et al., *Rehabilitation in Patients with Dementia Following Hip Fracture: A Systematic Review.* Physiotherapy Canada, 2012. **64**(2): p. 190-201.
38. Bachmann, S., et al., *Inpatient rehabilitation specifically designed for geriatric patients: systematic review and meta-analysis of randomised controlled trials.* BMJ, 2010. **340**: p. c1718.
39. Cameron, I., et al., *Geriatric rehabilitation following fractures in older people: a systematic review.* Health Technology Assessment, 2000. **4**(2): p. 105.
40. Cameron, I.D., *Models of rehabilitation -- commonalities of interventions that work and of those that do not.* Disability & Rehabilitation, 2010. **32**(12): p. 1051-1058.
41. Chong, C.P., J. Savige, and W.K. Lim, *Orthopaedic-geriatric models of care and their effectiveness.* Australasian Journal on Ageing, 2009. **28**(4): p. 171-176.

For peer review only - http://bmjopen.bmj.com/site/about/guidelines.xhtml

42. Drenth, J.C. and H. J.S.M., *Assessment of physical fitness using upper extremity tests in elderly patients during the non-weight bearing stage after hip fracture: a systematic review* [Dutch]. Nederlands Tijdschrift Voor Fysiotherapie, 2012. **122**(3): p. 104-110.
43. Egan M, J.S., Byrne K, Wells J, Stolee P, *Factors associated with a second hip fracture: a systematic review.* Clinical Rehabilitation 2008. **22**: p. 272-282.
44. Egol, K.A., K.J. Koval, and J.D. Zuckerman, *Functional Recovery Following Hip Fracture in the Elderly.* Journal of Orthopaedic Trauma, 1997. **11**(8): p. 594-599.
45. Green, J., et al., *Burden and medical needs in older patients with hip fractures and muscle atrophy or weakness.* Osteoporosis International, 2012. **23**: p. S93-S94.
46. Hu, F., et al., *Preoperative predictors for mortality following hip fracture surgery: A systematic review and meta-analysis.* Injury, 2012. **43**(6): p. 676-685.
47. Jelicic, M., G.I.J. Kempen, and L.M. van Eijk, *Do psychosocial factors affect recovery from hip fracture in the elderly? A review of the literature.* Journal of Rehabilitation Sciences, 1996. **9**(3): p. 77-81.
48. Mauffrey, C., *The management of subcapital fractures in the elderly population.* European Journal of Orthopaedic Surgery & Traumatology, 2009. **20**(5): p. 359-364.
49. Muir, S.W. and A.M. Yohannes, *The Impact of Cognitive Impairment on Rehabilitation Outcomes in Elderly Patients Admitted with a Femoral Neck Fracture: A Systematic Review.* Journal of Geriatric Physical Therapy, 2009. **32**(1): p. 24-32.
50. Robinson, K.M., *Efficacy of home care rehabilitation interventions.* Annals of Long-Term Care, 2000. **8**(9): p. 68-71.
51. Romeo, R., et al., *Treatment and prevention of depression after surgery for hip fracture in older people: cost-effectiveness analysis.* Journal of Affective Disorders, 2011. **128**(3): p. 211-9.
52. Visschedijk, J., et al., *Fear of falling after hip fracture: a systematic review of measurement instruments, prevalence, interventions, and related factors.* Journal of the American Geriatrics Society, 2010. **58**(9): p. 1739-1748.
53. Wang, X. and L.J. Emery, *Cognitive status after hip replacement.* Physical & Occupational Therapy in Geriatrics, 2002. **21**(1): p. 51-64.
54. *Scottish Intercollegiate Guidelines Network (SIGN) Management of hip fracture in older people: a national clinical guideline.* Edinburgh, SIGN, 2010.
55. Hip Fractures. October 2014. Agency for Healthcare Research and Quality, R., MD. http://www.ahrq.gov/research/findings/evidence-based-reports/hipfractp.html.
56. *Health Quality Ontario; Ministry of Health and Long-Term Care. Quality-Based Procedures: Clinical Handbook for Hip Fracture. Toronto, ON: Health Quality Ontario; 2013 May. 97 p. Available from: http://www.hqontario.ca/evidence/publications-and-ohtac-rec.*
57. *Minimum Standards for the Management of Hip Fracture in the Older Person. Agency for Clinical Innovation, New South Wales, Austrailia. Available at http://www.aci.health.nsw.gov.au/__data/assets/pdf_file/0004/222727/Minimum-Standards-for-the-Management-of-Hip-Fractures.pdf.*
58. *New Zealand Guidelines Group (2003). Acute management and immediate rehabilitation after hip fracture amongst people ages 65 years and over. Wellington, New Zealand. Available at http://www.moh.govt.nz/NoteBook/nbbooks.nsf/0/BF485CCA409F38C5CC256DCE0070F7B5/$file/Hip_Fracture_Management.pdf.*
59. Resnick, B., et al., *Adherence to an Exercise Intervention Among Older Women Post Hip Fracture.* Journal of clinical sport psychology, 2008. **2**(1): p. 41-56.
60. Resnick, B. and M.P. Daly, *The effect of cognitive status on outcomes following rehabilitation.* Family medicine, 1997. **29**(6): p. 400-405.
61. Resnick, B., et al., *Evaluating the components of the Exercise Plus Program: rationale, theory and implementation.* Health Education Research, 2002. **17**(5): p. 648-658.

For peer review only - http://bmjopen.bmj.com/site/about/guidelines.xhtml

62. Resnick, B., et al., *The Relationship Between Psychosocial State and Exercise Behavior of Older Women 2 Months After Hip Fracture.* Rehabilitation Nursing, 2007. **32**(4): p. 139-149.
63. Resnick, B., et al., *The Exercise Plus Program for Older Women Post Hip Fracture: Participant Perspectives.* The Gerontologist, 2005. **45**(4): p. 539-544.
64. Resnick, B., et al., *Testing the effectiveness of the exercise plus program in older women post-hip fracture.* Annals of Behavioral Medicine, 2007. **34**(1): p. 67-76.
65. Pearson M, H.H., Cooper C, Shepperd S, Pawson R, Anderson R, *Intermediate care: a realist review and conceptual framework.*, in *NIHR Service Delivery and Organisation Programme*2013.
66. Ritzer, G., *Meta-theorizing in Sociology.*1991, Lexington, MA: Lexington Books.
67. Roen, K., et al., *Extending systematic reviews to include evidence on implementation: Methodological work on a review of community-based initiatives to prevent injuries.* Social Science & Medicine, 2006. **63**(4): p. 1060-1071.
68. Pluye P, Robert E, and e.a. Cargo M, *Proposal: A mixed methods appraisal tool for systematic mixed studies reviews.* 2011.
69. Ritchie, J.S., L, *Qualitative data analysis for applied policy research.*, in *Analysing Qualitative Data* B.A.B. R.G, Editor 1994, Routledge: London. p. 173–194.
70. Bellelli, G., et al., *Action Observation Treatment Improves Recovery of Postsurgical Orthopedic Patients: Evidence for a Top-Down Effect?* Archives of Physical Medicine & Rehabilitation, 2010. **91**(10): p. 1489-1494.
71. Saltvedt, I., et al., *Development and delivery of patient treatment in the Trondheim Hip Fracture Trial. A new geriatric in-hospital pathway for elderly patients with hip fracture.* BMC Research Notes, 2012. **5**: p. 355.
72. Rolland, Y., et al., *Rehabilitation outcome of elderly patients with hip fracture and cognitive impairment.* Disability & Rehabilitation, 2004. **26**(7): p. 425-31.
73. Stenvall, M., et al., *Improved performance in activities of daily living and mobility after a multidisciplinary postoperative rehabilitation in older people with femoral neck fracture: a randomized controlled trial with 1-year follow-up.* Journal of Rehabilitation Medicine (Stiftelsen Rehabiliteringsinformation), 2007. **39**(3): p. 232-238.
74. Young, Y. and B. Resnick, *Don't worry, be positive: improving functional recovery 1 year after hip fracture.* Rehabilitation Nursing, 2009. **34**(3): p. 110-117.
75. Jackson, J. and J. Schkade, *Occupational adaptation model versus biomechanical-rehabilitation model in the treatment of patients with hip fractures.* American Journal of Occupational Therapy, 2001. **55**(5): p. 531-7.
76. Godfrey, M. and J. Townsend, *Older People in Transition From Illness to Health: Trajectories of Recovery.* Qualitative Health Research, 2008. **18**(7): p. 939-951.
77. Manthorpe, J. and M. Cornes, *Intermediate Care: Older People's Involvement and Experiences.* Journal of Integrated Care, 2004. **12**(6): p. 43-48.
78. Crotty, M., et al., *Early discharge and home rehabilitation after hip fracture achieves functional improvements: a randomized controlled trial.* Clinical Rehabilitation, 2002. **16**(4): p. 406-13.
79. Sirkka, M. and I. Bränholm, *Consequences of a hip fracture in activity performance and life satisfaction in an elderly Swedish clientele.* Scandinavian Journal of Occupational Therapy, 2003. **10**(1): p. 34-39.
80. Ziden, L., C. Wenestam, and M. Hansson-Scherman, *A life-breaking event: early experiences of the consequences of a hip fracture for elderly people.* Clinical Rehabilitation, 2008. **22**(9): p. 801-11.
81. Bauerle, D., N. Specht-Leible, and E. Voss, *Hip fracture - Changes in need of help and care - Cluster analysis - Formal and informal support. [German] Veranderungen des hilfe- Und pflegebedarfs nach huftnahen frakturen im hoheren lebensalter.* Zeitschrift fur Gerontologie und Geriatrie, 2004. **37**(5): p. 351-353.

For peer review only - http://bmjopen.bmj.com/site/about/guidelines.xhtml

82. Huang, T. and G. Acton, *Ways to maintain independence among Taiwanese elderly adults with hip fractures: a qualitative study.* Geriatric Nursing, 2009. **30**(1): p. 28-35.
83. Lieberman, D. and M. Friger, *Inpatient rehabilitation outcome after hip fracture surgery in elderly patients: a prospective cohort study of 956 patients.* Archives of Physical Medicine & Rehabilitation, 2006. **87**(2): p. 167-171.
84. McMillan, L., et al., *A grounded theory of taking control after fall-induced hip fracture.* Disability & Rehabilitation, 2012. **34**(26): p. 2234-2241.
85. Resnick, B., et al., *Factors that influence exercise activity among women post hip fracture participating in the Exercise Plus Program.* Clin Interv Aging, 2007. **2**(3): p. 413-27.
86. Bandura, A., *Self-efficacy: The exercise of control*1997, New York, NY, US: W H Freeman/Times Books/ Henry Holt & Co. ix, 604.
87. Morrison, V.L., et al., *Improving Emotional Outcomes following Acute Stroke: A Preliminary Evaluation of a Work-Book Based Intervention.* Scottish Medical Journal, 1998. **43**(2): p. 52-53.
88. Johnston, M., et al., *Recovery from disability after stroke as a target for a behavioural intervention: Results of a randomized controlled trial.* Disability and Rehabilitation, 2007. **29**(14): p. 1117-1127.
89. Williams, N.H., et al., *Fracture in the Elderly Multidisciplinary Rehabilitation (FEMuR): study protocol for a phase II randomised feasibility study of a multidisciplinary rehabilitation package following hip fracture [ISRCTN22464643].* Pilot and Feasibility Studies, 2015. **1**(1): p. 1-22.
90. Pearson, M., et al., *Using realist review to inform intervention development: methodological illustration and conceptual platform for collaborative care in offender mental health.* Implement Sci, 2015. **10**: p. 134.
91. Taylor, N.F., Harding, K.E., Dowling, J., Harrison, G, *Discharge planning for patients receiving rehabilitation after hip fracture: A qualitative analysis of physiotherapists' perceptions.* Disability and Rehabilitation, 2010. **32**(6): p. 492-499.
92. Taylor, N.F., Barelli, C and Harding, K.E, *Community ambulation before and after hip fracture: a qualitative analysis.* Disability and Rehabilitation, 2010. **32**(15): p. 1281-90.
93. McMillan, L., et al., *'Balancing risk' after fall-induced hip fracture: the older person's need for information.* International Journal of Older People Nursing, 2014. **9**(4): p. 249-257.
94. Long, A.F., R. Kneafsey, and J. Ryan, *Rehabilitation practice: challenges to effective team working.* International Journal of Nursing Studies, 2003. **40**(6): p. 663-673.
95. Williams, N.H., et al., *Fracture in the Elderly Multidisciplinary Rehabilitation (FEMuR): a phase II randomised feasibility study of a multidisciplinary rehabilitation package following hip fracture.* BMJ Open, 2016. **6**(10): p. e012422.

Figure legends:

Fig 1. Development of programme theories for informing the content of the enhanced rehabilitation intervention.

Fig. 2. Frequency of rehabilitation visits following hip fracture

Fig. 3. Proposed outcomes of individual intervention components target specific programme theories.

For peer review only - http://bmjopen.bmj.com/site/about/guidelines.xhtml

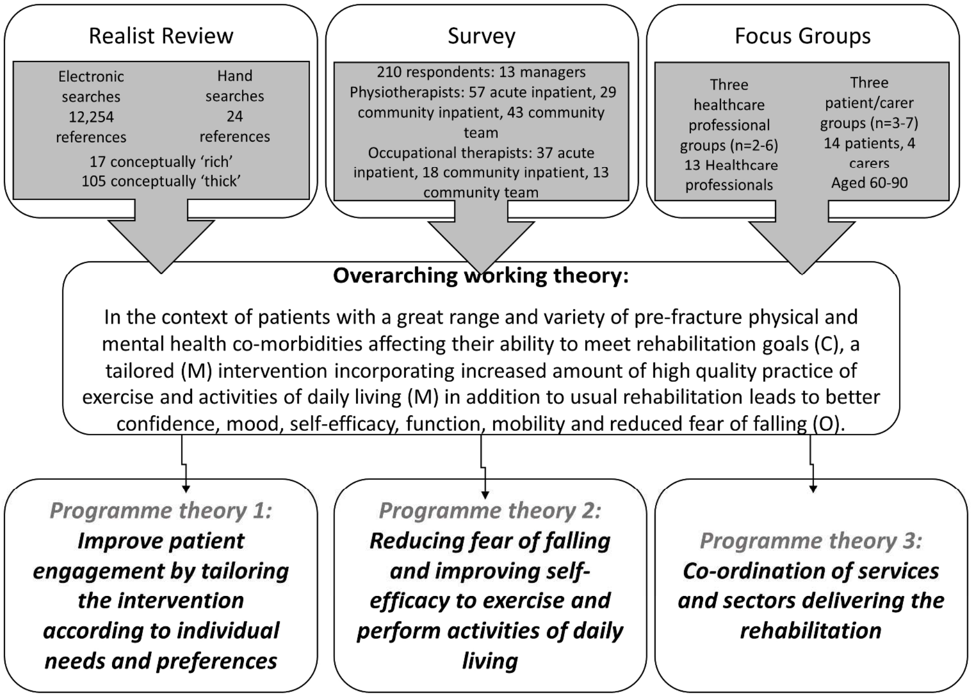

Fig 1. Development of programme theories for informing the content of the enhanced rehabilitation intervention.

254x190mm (300 x 300 DPI)

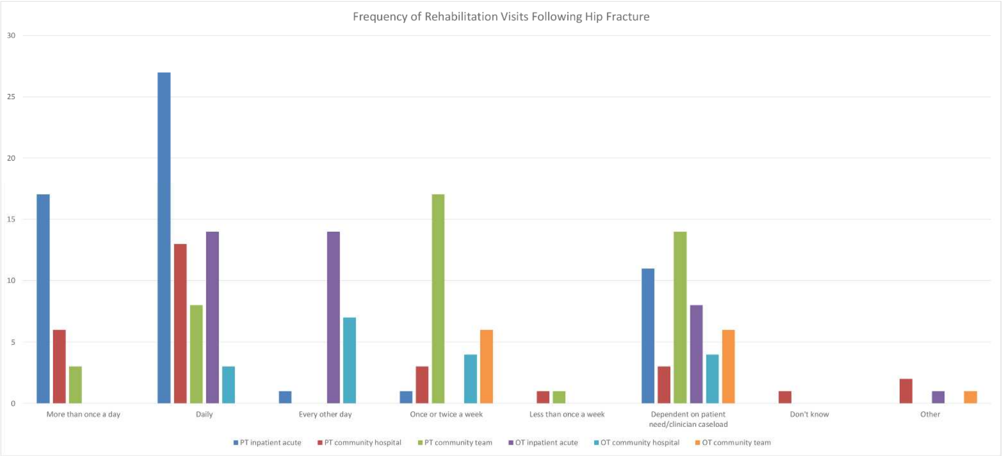

Fig. 2. Frequency of rehabilitation visits following hip fracture

345x157mm (300 x 300 DPI)

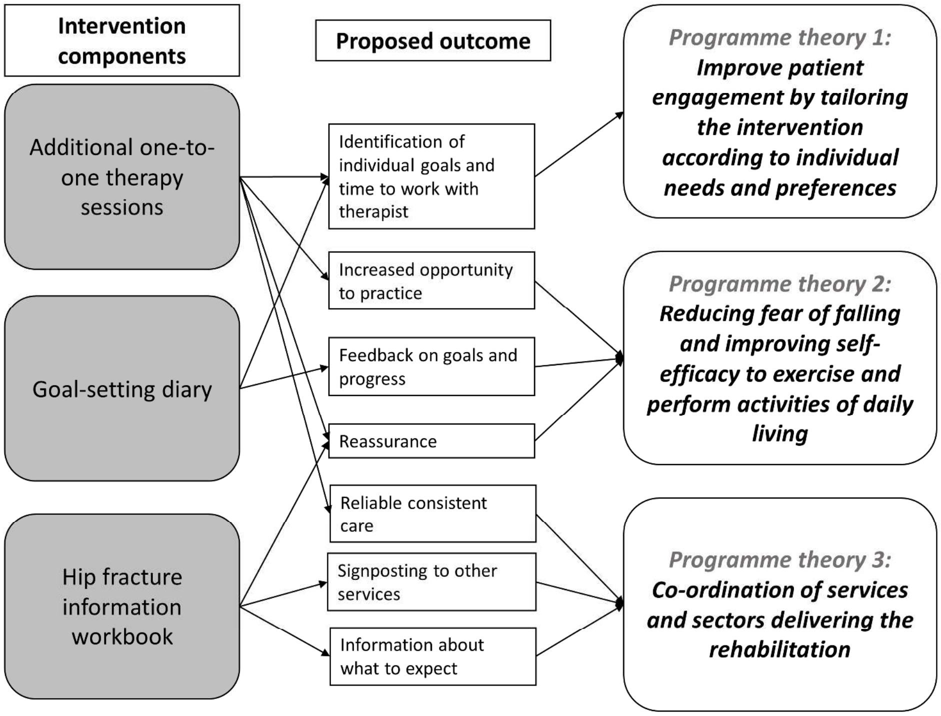

Fig. 3. Proposed outcomes of individual intervention components target specific programme theories.

254x190mm (300 x 300 DPI)

Appendix 1 – List of databases searched for realist review.

- MEDLINE
- MEDLINE In-Process & Other Non-Indexed Citations
- OLDMEDLINE
- EMBASE
- Cumulative Index to Nursing and Allied Health Literature (CINAHL)
- Allied and Complimentary Medicine Database (AMED)
- British Nursing Index
- Health Management Information Consortium (HMIC)
- PsychINFO
- Cochrane Central Register of Controlled Trials (CENTRAL)
- Database of Abstracts of Reviews of Effects (DARE)
- Cochrane Database of Systematic Reviews (CDSR)
- Health Technology Assessment (HTA) Database
- NHS Economic Evaluation Database (NHS EED)
- Science Citation Index
- Social Science Citation Index (SSCI)
- Index to Scientific & Technical Proceedings (ISTP)
- Physiotherapy Evidence Database (PEDro)
- BIOSIS
- System for Information on Grey Literature In Europe (SIGLE)
- Web of Knowledge Index of Theses and Dissertations

For peer review only

BMJ Open

Development of an evidence-based complex intervention for community rehabilitation of hip fracture patients using realist review, survey and focus groups

Journal:	*BMJ Open*
Manuscript ID	bmjopen-2016-014362.R2
Article Type:	Research
Date Submitted by the Author:	16-Aug-2017
Complete List of Authors:	Roberts, Jessica; Bangor University, North Wales Centre for Primary Care Research<br>Din, Nafees; Bangor University, North Wales Centre for Primary Care Research<br>Williams, Michelle; Bangor University<br>Hawkes, Claire; University of Warwick Warwick Medical School, Clinical Trials Unit<br>Charles, Joanna; Bangor University, Centre for Health Economics & Medicines Evaluation<br>Hoare, Zoe; Bangor University, North Wales Organisation for Randomised Trials in Health<br>Morrison, Val; School of Psychology, Bangor University<br>Alexander, Swapna; Betsi Cadwaladr University Health Board<br>Lemmey, Andrew; Bangor University, School of Sports, Health and Exercise Science<br>Sackley, Catherine; Kings College London,<br>Logan, Phillipa; The University of Nottingham, Community Health Sciences<br>Wilkinson, Clare; Bangor University, 9. North Wales Centre for Primary Care Research<br>Rycroft-Malone, Jo; Bangor University, School of Healthcare Sciences;<br>Williams, Nefyn; Bangor University, North Wales Organisation for Randomised Trials in Health
**Primary Subject Heading**:	Rehabilitation medicine
Secondary Subject Heading:	Research methods, Health services research, Qualitative research
Keywords:	Hip < ORTHOPAEDIC & TRAUMA SURGERY, realist review, survey, focus groups, intervention development, Rehabilitation medicine < INTERNAL MEDICINE

SCHOLARONE™
Manuscripts

Development of an evidence-based complex intervention for community rehabilitation of hip fracture patients using realist review, survey and focus groups

Jessica L Roberts PhD[1], Nafees Ud Din MSc MFDS RCS[1], Michelle Williams BA[1], Claire A Hawkes PhD RN[4], Joanna M Charles PhD[1], Zoe Hoare PhD[1], Val Morrison PhD CPsychol[3], Swapna Alexander MD FRCP[2], Andrew Lemmey PhD[5], Catherine Sackley PhD[6], Phillipa Logan PhD[7], Clare Wilkinson MD FRCGP[1], Jo Rycroft-Malone PhD[1], Nefyn H Williams PhD FRCGP[1,2.]

[1]School of Healthcare Sciences, Bangor University, UK

[2]Betsi Cadwaladr University Health Board, North Wales, UK

[3]School of Psychology, Bangor University, UK

[4]Warwick Clinical Trials Unit, University of Warwick, UK

[5]School of Sports, Health and Exercise Sciences, Bangor University, UK

[6] Division of Health and Social Care Research, King's College, London, UK

[7]School of Medicine, University of Nottingham, UK

Corresponding author: Dr Jessica Roberts, North Wales Centre for Primary Care Research, Bangor University, Cambrian 2, Wrexham Technology Park, Wrexham LL13 7YP, UK.

Tel: +44 (0)1248 383516 e-mail: j.l.roberts@bangor.ac.uk

For peer review only - http://bmjopen.bmj.com/site/about/guidelines.xhtml

Abstract

Objectives: To develop an evidence and theory based complex intervention for improving outcomes in elderly patients following hip fracture.

Design: Complex-intervention development (Medical Research Council [MRC] framework phase I) using realist literature review, surveys and focus groups of patients and rehabilitation teams.

Setting: North Wales

Participants: Surveys of therapy managers (n=13), community and hospital-based physiotherapists (n=129) and occupational therapists (n=68) throughout the UK. Focus groups with patients (n=13), their carers (n=4) and members of the multidisciplinary rehabilitation teams in North Wales (n=13).

Results: The realist review provided understanding of how rehabilitation interventions work in the real world context and three programme theories were developed: improving patient engagement by tailoring the intervention to individual needs; reducing fear of falling and improving self-efficacy to exercise and perform activities of daily living; and co-ordination of rehabilitation delivery. The survey provided context about usual rehabilitation practice; focus groups provided data on the experience, acceptability and feasibility of rehabilitation interventions.

An intervention to enhance usual rehabilitation was developed to target these theory areas comprising: a physical component consisting of six additional therapy sessions; and a psychological component consisting of a workbook to enhance self-efficacy and a patient held goal-setting diary for self-monitoring.

For peer review only

Conclusions: A realist approach may have advantages in the development of evidence-based interventions and can be used in conjunction with other established methods to contribute to the development of potentially more effective interventions. A rehabilitation intervention was developed which can be tested in a future randomised controlled trial (MRC framework phases II and III).

Review registration: PROSPERO 2012:CRD42012003208.

Article summary

Strengths and limitations of this study:

- A complex intervention for hip fracture rehabilitation was developed, which was evidence based and theoretically underpinned (Medical Research Council [MRC] framework phase I).
- Programme theories were developed from a realist review of the literature. A survey added context, and focus groups provided data on the experience, acceptability and feasibility of rehabilitation interventions.
- The methods used to develop this rehabilitation programme may be applicable to the development of other complex interventions.
- The feasibility and acceptability of the developed intervention are reported separately, but evidence of effectiveness and cost-effectiveness requires testing in a randomised controlled trial (MRC framework phases II and III).

For peer review only

For peer review only - http://bmjopen.bmj.com/site/about/guidelines.xhtml

Background

Proximal femoral fracture, more commonly referred to as hip fracture, is a common, major health problem in the elderly [1]. It is associated with prior fragility fracture, and other co-morbidities such as: cognitive impairment, under-nutrition, decreased bone mineral density, frailty, poor physical functioning, vision problems and weight loss [2]. Mortality is high with 14-58% of patients dying within 12 months [3, 4] and up to 53% do not regain their previous level of functioning [5, 6]. Proximal femoral fractures cost the UK economy approximately £2.3 billion a year [7]. Management guidelines from the National Institute of Health and Care Excellence (NICE) [8] recommend multidisciplinary rehabilitation, which has the potential to maximise recovery, enhance quality of life and maintain independence. However, systematic reviews conclude that there is insufficient evidence of overall effectiveness or cost-effectiveness, but individual components of such programmes may show promise [9-11].

Rehabilitation programmes for hip fracture are complex interventions due to their multi-faceted nature and the involvement of many heterogeneous factors including individual patient circumstances and co-morbidities, healthcare professionals, rehabilitation setting and social influences [12]. The interaction of these factors in the real world and how they interplay and influence each other to determine the success and failure of such programmes is poorly understood, making it difficult to identify which specific components of rehabilitation programmes are effective and under what circumstances [13, 14]. Whilst there have been many systematic reviews of hip fracture rehabilitations [5, 10, 11], these are only able to evaluate the evidence of whether an intervention works and do not allow for exploration of how and why an intervention leads to its reported outcomes. Realist

For peer review only - http://bmjopen.bmj.com/site/about/guidelines.xhtml

reviews aim to elucidate the mechanisms behind an intervention and determine 'what works, for whom, in what circumstances, and why?' whilst taking into account the heterogeneous nature of such interventions and the settings in which they are delivered [15]. This involves multiple steps, which starts with extracting working theories from individual studies and developing them into 'programme theories' which describe what programmes or interventions are expected to do and how they are intended to work. These are compared and contrasted to develop intermediate programme theories, which refer to propositions of how a programme is likely to produce intended outcomes. These are then tested and refined into a final list of theories, which describe the mechanism or causal force that makes things happen in certain circumstances or contexts, such as patient characteristics or place of rehabilitation, which result in desired outcomes [13].

Realist reviews provide a flexible way of exploring causal relationships, thus aiding our understanding of intervention mechanisms and supporting the development of potentially more effective interventions [16, 17].

We therefore undertook a realist review of the available evidence for hip fracture rehabilitation to develop theory on the context, mechanism and outcomes of existing rehabilitation programmes, with this forming the basis for the development of our own evidence-based intervention for subsequent testing in a feasibility study [18]. The development of these theory areas was performed in conjunction with a survey of current practice by UK rehabilitation health professionals and focus groups with patients, carers and multidisciplinary rehabilitation team. Whilst the MRC framework for complex interventions provides general guidelines for intervention development and supports the use of theoretical underpinning [12], detailed guidance on how this framework is practically

For peer review only

applied to intervention development is lacking. To contribute to bridging this knowledge gap, this paper sets out the methodology of how the evidence base was established and utilised for intervention development, linking the findings of the review, survey and focus groups to the proposed aims of our intervention and how we expected these to facilitate our intended outcomes.

Methods

A summary of the methods used is presented below. Further detail can be found in the final report to the funder. The development of the community-based rehabilitation package was informed by three complementary work packages. A coherent theoretical basis for the intervention was developed from a realist literature review. Initial findings from the review were used to develop the questions for a survey. A survey of current services described usual practice, and was an additional source of relevant theories that contributed to the realist review. The initial findings from the review were also used to develop topic guides for the focus groups and the initial framework for their analysis. Focus groups provided data on the experience, acceptability and feasibility of rehabilitation interventions.

Realist review

A realist review was used to identify the evidence base and develop a theoretical understanding. A scoping search of systematic reviews [5, 10, 11, 19-53], guidelines [8, 54-58] and theoretically rich primary studies [59-64] was performed to map out the important areas and research gaps (NUD, NHW). This generated a list of questions, which could be

For peer review only - http://bmjopen.bmj.com/site/about/guidelines.xhtml

For peer review only

grouped under different domains relating to: patients, healthcare and rehabilitation teams, rehabilitation programmes, and the settings in which rehabilitation was delivered.

The list of questions were formulated into statements that described how these different domains interact. These statements were subsequently refined during discussions between members of the research team (JR-M, NUD, NHW, JMC) and with other researchers from the School of Healthcare Sciences, Bangor University, in two realist review workshops.

Feedback from experts in health psychology, rehabilitation and implementation research were combined with initial survey and focus group findings into candidate programme theories. These intermediate working theories were used as the basis for bespoke data extraction forms.

Literature search

The literature search strategy used by the NICE guideline review of multidisciplinary rehabilitation programmes for hip fracture [8] was adapted for this review, with no search filters for study design; this intentional inclusivity enabled review of different types of study, which, in turn, facilitated formulation and examination of the emerging theories. Twenty one databases were searched from inception to Feb 2013 in order to be used as the next step of programme development (Appendix 1). Citation tracking and internet search engines were used to identify additional evidence as the review progressed and as new ideas emerged. Materials were retrieved purposively to answer specific questions or test specific theories until no new themes emerged.

For peer review only - http://bmjopen.bmj.com/site/about/guidelines.xhtml

Screening and categorisation of references

Participants of interest were elderly adults with proximal hip fracture. The intervention of interest was multidisciplinary rehabilitation following proximal hip fracture. Outcomes of interest were mortality, pain, functional status, quality of life, health utility, health service use, costs, and patients' experiences. A working definition of multidisciplinary rehabilitation was adapted from a review of intermediate care services [65] (Box 1). Separate reviewers screened identified studies for relevance and discrepancies were resolved after discussion (NUD, NHW, JC). Potentially relevant papers were categorised according to study type, and then according to whether they were conceptually 'rich', 'thick' or 'thin' [66, 67] (NUD, NHW). We started by extracting data from all of the 'rich' studies and sampled data from the conceptually 'thick' studies until saturation was reached. The purpose of this was to make the database manageable and to build and examine theories from studies with the most relevant concepts.

For peer review only - http://bmjopen.bmj.com/site/about/guidelines.xhtml

For peer review only

Box 1: Working definition of multidisciplinary rehabilitation used to screen sources of evidence

Purpose	Supports re-enablement of the frail elderly (over 65 years old) following proximal hip fracture to achieve their functional potential and maintain independent living where possible
Functions	A bridge between: a) the hospital and the community;<br>b) different healthcare sectors and personal social care<br>Views people holistically<br>Time limited (up to one year following fracture)
Structure	Teams based in hospitals, the community or across both sectors
Content	Treatment and therapy (to increase strength, confidence and ability to perform activities of daily living)<br>Psychological, practical and social support<br>Support/training to develop skills and strategies for self-management
Delivery	Care delivered by a multidisciplinary team or teams

Data extraction and quality assessment

Data were extracted by one reviewer (NUD) and checked for accuracy by a second (NHW). We assessed study quality using the mixed methods appraisal tool (MMAT) [68]. Data from effectiveness studies were exported into structured tables to show the strength and direction of the treatment effects.

For peer review only - http://bmjopen.bmj.com/site/about/guidelines.xhtml

Testing the theories with quantitative and qualitative evidence

Theories were refined through an iterative process comparing individual study programme theories in turn. Data for each individual study were examined in terms of the identified programme theories and the interaction between mechanisms, context and outcomes, starting with data extracted from studies that were conceptually 'rich' and continuing with those that were conceptually 'thick'. The data were then examined across the different studies to detect patterns and themes. A second set of refined date extraction forms were used to test each theory in turn, and adjudicate between components of the final programme theories.

Survey of UK Hip Fracture Centres

Survey development

A UK wide web-based survey was conducted, targeting physiotherapists, occupational therapists and hip fracture centre therapy service managers working in the rehabilitation of patients over 65 years of age who have had surgery for proximal hip fracture. NICE guidance on hip fracture rehabilitation [5] was used with initial findings from the scoping review as the starting point for developing the questions. The questionnaires were piloted on members of staff across one health board in Wales, and minor amendments were made.

Data collection

Three versions of the survey were developed for hip fracture centre managers, physiotherapists and occupational therapists. The managers' survey focused on the organisation of services, whilst the therapists' questionnaires focussed on aspects of clinical practice such as session content, frequency and location, and how assessments were

For peer review only - http://bmjopen.bmj.com/site/about/guidelines.xhtml

conducted. The therapists' versions were further subdivided according to healthcare setting: acute hospital, community hospital or community based team.

The survey was open for seven weeks from 06/08/2013 to 25/09/2013. We surveyed a sample of senior managers who had a strategic role in rehabilitation services for this group of patients and aimed to achieve a 10% sample of all UK centres performing hip fracture surgery. Centres in Wales, Northern Ireland and England were identified from publically available information on the National Hip Fracture Database (NHFD). Hospitals in Scotland were contacted separately and directly. We purposively sampled for geographic spread and centre size, contacting centres by telephone and through advertising on the NHFD. Twenty four centres from across the UK agreed to take part.

Data Analysis

Descriptive statistics were used to provide frequency (counts, percentages) data concerning current services and practice, where the answer format provided pre-determined response options (ZH). Where the response format was open-ended, responses were coded and categorised into themes (MW). The integrated care pathways and physiotherapy exercise sheets returned to the team were qualitatively reviewed to provide description of commonalities and differences (MW).

Focus Groups

Focus groups were completed at the three acute hospital sites across North Wales within Betsi Cadwaladr University Health Board (BCUHB). Three focus groups of members of the multidisciplinary rehabilitation teams in the community and the hospital, and four focus

For peer review only

For peer review only - http://bmjopen.bmj.com/site/about/guidelines.xhtml

groups for patients and their carers were organised. Informed consent procedures were followed for recruitment, as approved by UK NHS North Wales Research Ethics Committee.

People who were over 65 years, were receiving rehabilitation following surgical repair of a proximal hip fracture within the last 3-12 month, were living independently prior to fracture, and were able to provide informed consent were eligible to take part.

Eligible participants were identified from the NHFD, through the medical and nursing staff who were responsible for maintaining the database at each site.

Data collection

Discussions were semi-structured and run by a moderator (CH) and co-moderator (MW, NHW, JLR or NUD) using a topic guide containing open-ended questions regarding experiences, perceptions and beliefs about rehabilitation following proximal hip fracture. In the professionals' focus groups, patient scenarios were also used to stimulate discussion about the sort of rehabilitation patients would be likely to receive. In the later patient focus groups, we explored initial ideas for the intervention to gain feedback. The focus group discussions were digitally recorded and fully transcribed into the speaker's original language, with any portions in Welsh subsequently translated into English for analysis. The interviews were thematically analysed using the Framework approach [69]. The initial framework used was broadly developed from the theory areas identified as important to guide the realist review and it was used to index the transcripts. The researcher developed an initial interpretation of the data using the framework, and grouped the data into themes which were reviewed by a second researcher experienced in framework analysis. A third researcher reviewed the initial framework, original transcripts and the draft analysis to

For peer review only - http://bmjopen.bmj.com/site/about/guidelines.xhtml

For peer review only

make final decisions on theme structure and content. The initial and third researchers agreed the final analysis.

Development of the intervention

The final programme theories and the results from the survey and the initial focus groups were discussed by all of the research team in order to identify the important components of the rehabilitation intervention. The intervention components were discussed and refined in the final focus groups.

For peer review only

For peer review only - http://bmjopen.bmj.com/site/about/guidelines.xhtml

Results

A summary of the results is presented below. Further detail can be found in the final report to the funder [18]. The literature review, survey results and qualitative data were used to develop the following overarching working theory;

> "In the context of patients with a great range and variety of pre-fracture physical and mental health co-morbidities affecting their ability to meet rehabilitation goals, a tailored intervention incorporating increased quality and amount of practice of exercise and activities of daily living in addition to usual rehabilitation leads to better confidence, mood, self-efficacy, function, mobility and reduced fear of falling."

This was then broken down into three component programme theories described below.

(Figure 1).

Programme theory 1: Improve patient engagement by tailoring the intervention according to individual needs and preferences

Elderly proximal hip fracture patients presenting with a range of pre-fracture physical and mental functioning and a variety of co-morbidities need a rehabilitation programme that is tailored to individual needs in order to achieve appropriate outcomes such as improved physical functioning, greater mobility, reduced disability and independent living.

Findings from the realist review indicated that tailoring of patient care requires a detailed assessment of patients' pre-fracture level of functioning [70, 71], current cognitive status [72] and other co-morbid conditions [73, 74]. It should also involve collaborative decision making through discussion and agreement with patients, their family and carers regarding:

For peer review only - http://bmjopen.bmj.com/site/about/guidelines.xhtml

realistic and achievable [74], but modifiable [75], short-term and longer term goals of rehabilitation [76, 77], the most appropriate setting for rehabilitation suited to patients' needs and abilities [78-80], and any adaptation of the physical environment to facilitate day to day activities [80, 81]. In addition, the provision of enhanced support through active engagement of carers and rehabilitation professionals to motivate and facilitate the regular practice of exercises and activities of daily living [62, 63], improve health perceptions [82], address and adjust outcome expectations [63, 83], and address information needs [63, 64, 84].

In the survey, tailored rehabilitation was also identified as an important aspect of service provision. Respondents from all categories reported that the frequency of rehabilitation visits received were influenced by individual patient need (Fig 2.). Survey findings revealed that routine clinical practice was broadly in line with current guidance, but variability existed in the provision of services, especially in the community. Variation was reported in the frequency of rehabilitation visits following hip fracture (Fig. 2), with some services performing multiple visits a day and others visiting patients less than once a week. There was also variation in the types of activities included. For example, whilst the majority of occupational therapists (over 95%) included prescribing equipment and practising activities of daily living in their usual activities, less than 50% included anxiety management and developing self-awareness. The importance of tailored care was also highlighted in focus groups (Table 1), with many patients finding it hard to engage in strengthening exercises if they were not part of an individualised plan that focussed on personal goals. The first emergent theme from the focus groups related to the variability of care provision, which was partly because of individual tailoring of treatment, but also geographical variation in the

For peer review only - http://bmjopen.bmj.com/site/about/guidelines.xhtml

availability of resources. Furthermore, co-morbidities and pre-fracture functioning determined what patients were able to do and affected their attitude to exercise, which could be taken into account through individual tailoring of care plans.

Table 1. Focus group themes and supporting quotes from patients, carers and healthcare professionals

Theme	Supporting quotes
**Variation of rehabilitation care provision**	*"It depends completely on the patient you can't just say well this is what is going to happen to every patient, they vary so much… there is different avenues depending on what they present"* R4, community hospital physiotherapist, FG1121.<br><br>*"It depends on what, what procedure she [the surgeon] has done to fix the fractured NOF [neck of femur] as to what level of interventions we do"* R2, occupational therapist, FG1321.<br><br>*"You are dealing with very angry relatives who were under the presumption that because they are under our service, that they will automatically get care and they won't, not unless there is a need"* R1, clinical specialist physiotherapist, FG1321.
**Facilitators and barriers to rehabilitation**	*"There's a limit to what you can do at home, I got to the stage where I needed equipment… the first time I went to the gym and saw the physio there, I thought yes...It hurt, it was painful, but at least I felt I'm sure I'm going to get somewhere, and it has it's been brilliant."* F2, female patient, FG1111<br><br>*"We refer a lot [to falls group], as long as they can get transport"* (R, FG1221).<br><br>*"Seeing the physio, it's a mixture of more exercises and going through it but also it's the ability just to have someone to talk through things like what to do with the pain"* M1, male patient, FG1111

For peer review only - http://bmjopen.bmj.com/site/about/guidelines.xhtml

**Psychosocial impact of hip fracture**	*"Couple of women recently and have taken ages, whereas initially talking to them they are women you know sort of retired but really active, do loads, but then they have fallen and really I think it's more, you know the shock of the falling over and not being able to do things it does take them quite a long time to get over it."* R3, acute hospital occupational therapist, FG1121.<br><br>*"You think you are going to fall all the time, erm… so it is just practice I think, just keep doing it, keep doing little bits and erm…I had the reassurance from the physiotherapist who said 'no, by next summer you will be doing exactly what you were doing last summer.'"* R1, female patient, FG1212.<br><br>*"It's to do with personal care as well, and to raise confidence as well, that's a lot to do with it because people who have had the falls, it's their confidence really that's taken a big knock"* R3, reablement team, FG1221.
**Need for information**	*"I didn't know what to do I didn't know whether to sit, and rest or try to exercise or what nobody told me anything… people don't explain… tell you so that you can understand. You just, left to ponder it over for yourself"* R3, female patient, FG1211.<br><br>*"There was a whole series of questions I had that had come up over the previous three weeks and I think the ability to go and talk to someone, with different experience and knowledge was very important for me now"* M1, male patient, FG1111<br><br>*"Care is good, communication is rubbish"* I3, male carer, FG1311.

For peer review only - http://bmjopen.bmj.com/site/about/guidelines.xhtml

Programme theory 2: Reducing fear of falling and improving self-efficacy to exercise and perform activities of daily living

Proximal hip fracture results in poor physical functioning, fear of falling, low mood and lack of self-efficacy requiring improved quality and increased amount of practice of physical exercises, activities of daily living and psychological tasks in order to gain mastery and control to improve confidence, mobility and physical functioning.

Enhancing the practice and quality of exercise and activities of daily living has both physical and psychological components [63, 64]. This consists of supervision and coaching by health professionals in order to improve skills and confidence to promote independent and unsupervised practice [80], with resulting increases in the duration, frequency and quality of exercises for improving strength, balance, gait and activities of daily living. Addressing psychological concerns is also important, particularly to improve mood [59, 85]. Motivation to practise can be improved by setting appropriate, realistic goals and developing mechanisms for monitoring and providing feedback [85, 86].

According to the survey, more rehabilitation staff resources were needed to provide this support. Although patients' cognitive status, mood, self-efficacy and fear of falling were assessed; routine assessments using validated tools were not performed everywhere and the frequency that progress was assessed varied (Table 2). The importance of psychological factors was also highlighted in focus groups where a second emergent theme was facilitators and barriers to rehabilitation, one of which was the reliance on patient's own self-motivation to seek out and access services. The level of patient engagement in the rehabilitation programme depended upon its perceived relevance to their day-to-day activities, and in the absence of this the amount of practice was likely to decline. A third

For peer review only - http://bmjopen.bmj.com/site/about/guidelines.xhtml

Table 2. Validated measures reported to be used by physiotherapists and occupational therapists in acute and community settings.

	Acute hospital	Community Hospital	Community Team
**Assessment**	**Tools used**	**Tools used**	**Tools used**
**Cognitive status**	Abbreviated mental test score (AMTS).<br>Mini mental state examination (MMSE).<br>Rapid assessment test for delirium and cognitive impairment (4AT)<br>Six Item Cognitive Impairment Test (6CIT).<br>Montreal Cognitive Assessment (MoCA).<br>Addenbrooke's Cognitive Examination (ACE-R).	Six Item Cognitive Impairment Test (6CIT).<br>Montreal Cognitive Assessment (MoCA).<br>Abbreviated mental test score (AMTS).MMSE<br>Middlesex Elderly Assessment of Mental State (MEAMS).	Six Item Cognitive Impairment Test (6CIT).<br>Addenbrooke's Cognitive Examination (ACE-R).<br>Mini mental state examination (MMSE).<br>Middlesex Elderly Assessment of Mental State (MEAMS).<br>Montreal Cognitive Assessment (MoCA).<br>COG test<br>Rowland Universal Dementia Assessment Scale (RUDAS).
**Mood**	Hospital Anxiety and Depression Scale (HADS).<br>Geriatric Depression Scale (GDS).<br>Unified assessment proforma.<br>Montreal Cognitive Assessment (MoCA).	Geriatric Depression Scale (GDS).Hospital Anxiety and Depression Scale (HADS).<br>The 12-Item Short Form Health Survey (SF-12).<br>Canadian Occupational Performance Measure (COPM).	Six Item Cognitive Impairment Test (6CIT).<br>Geriatric Depression Scale (GDS).<br>Depression Scale (HADS).<br>Therapy Outcome Measure (TOM).
**Self-efficacy**	Unified assessment proforma.<br>10m walk.	The Falls Efficacy Scale (FES)<br>Visual Analogue Scale (VAS).<br>Canadian Occupational Performance Measure (COPM).	Euro Qol (EQ-5D).<br>Therapy Outcome Measure (TOM).
**Fear of falling**	Visual Analogue Scale (VAS).<br>Berg Balance Scale.<br>Elderly mobility scale.<br>Oxford hip scale.<br>Tinetti Assessment Tool.	Falls Risk Assessment Tool (FRAT).<br>Visual Analogue Scale (VAS).<br>The Falls Efficacy Scale (FES).<br>The Falls Efficacy Scale International (FES-I).<br>Tinetti Assessment Tool.<br>Canadian Occupational Performance Measure (COPM).	Falls Risk Assessment Tool (FRAT).Visual Analogue Scale (VAS).
**Health utility**		Euro Qol (EQ-5D).	Euro Qol (EQ-5D).

For peer review only - http://bmjopen.bmj.com/site/about/guidelines.xhtml

focus group theme was the psychosocial effects of the fracture, fear of falling in particular, which reduced confidence and increased the reliance on walking aids. This fear not only affected engagement in the rehabilitation programme but also impacted on wider social interactions, leading to feelings of isolation.

Programme theory 3: Co-ordination of services and sectors delivering the rehabilitation

The diversity of services provided by different disciplines, across sectors from a variety of funders requires a co-ordinated provision of the multidisciplinary rehabilitation programme in order to deliver appropriate physical, functional and psychological interventions to patients in a timely manner.

The co-ordination of multidisciplinary care from the acute hospital into the community, required good communication between rehabilitation professionals and careful discharge planning. Patients valued the help and support they received from healthcare teams during their recovery and regarded this as the single most important factor in their recovery, so the provision of consistent and reliable care was vital.

Most respondents in the survey from both acute and community hospital settings reported that routine clinical practice was following the latest NICE (2011) [8] and SIGN (2009) [54] guidance. Multidisciplinary teams working with common goals across settings were a strength, but there was variability in service provision, especially with regard to what was available in the community. Liaison between the acute hospital and the community could be improved, as could communication with patients and carers.

The fourth focus group theme was a need for more information for patients and their carers about what to expect following the hip fracture and how to access all of the available

For peer review only - http://bmjopen.bmj.com/site/about/guidelines.xhtml

resources. The complexity in programme provision and the often poor communication between different sectors, meant that rehabilitation was neither smooth nor seamless, and because of this lack of consistency, patients felt unsupported in their recovery. Patients and their carers required reassurance from qualified professionals about which activities were safe to perform in order to overcome these barriers, highlighting the role of the therapist as a mediator to improve their self-efficacy.

Designing a rehabilitation intervention

Considering these findings the rehabilitation intervention needed to:

- identify individual goals with help from a therapist;
- enhance self-efficacy;
- increase the opportunity to practise prescribed exercises and activities of daily living;
- support the self-monitoring of progress towards identified goals;
- give encouragement and support from professionals;
- provide information on what to expect during rehabilitation;
- provide reliable and consistent care;
- sign post to other available services.

In order to address these, we developed a rehabilitation programme comprising both physical and psychological components (Fig. 3). The physical component consisted of additional rehabilitation sessions, tailored to individual need, following discharge home. The psychological component consisted of a patient-held information workbook, developed using an existing stroke rehabilitation workbook [87, 88] as an exemplar, and

For peer review only - http://bmjopen.bmj.com/site/about/guidelines.xhtml

a goal-setting diary. These aimed to improve patient engagement in the rehabilitation programme by giving patients a sense of ownership of their own recovery.

The additional sessions were also an opportunity for patients to obtain reassurance and guidance from a qualified healthcare professional. Similarly, the outcome of the psychological components aimed to increase confidence and self-efficacy that would affect patient's ability and willingness to perform exercises, thus improving their physical outcomes.

A detailed logic model of the intervention activities, their proposed long and short-term goals and how these target different components of the International Classification of Functioning framework has previously been published [89], along with how the intervention addresses specific areas of existing NICE guidance for hip fracture rehabilitation.

Discussion

There were three programme theories from the realist review: improving patient engagement by tailoring the intervention according to individual needs and preferences; reducing fear of falling and improving self-efficacy to exercise and perform activities of daily living; and co-ordination of services and sectors delivering the rehabilitation. These were reflected in the survey data highlighting that whilst routine clinical practice was broadly in line with current guidance, there was variability in the provision of services, especially in the community, and that important psychological mediators such as self-efficacy and fear of falling were not routinely assessed using validated tools. They also agreed with the four focus group themes of: variation in rehabilitation care provided; the need for more information; facilitators and barriers to rehabilitation; and the psychosocial impact of hip

For peer review only - http://bmjopen.bmj.com/site/about/guidelines.xhtml

fracture. These findings informed the development of a community-based rehabilitation intervention consisting of a psychological component delivered using a workbook and a patient-held goal setting diary and a physical component comprising additional rehabilitation sessions.

Other studies have acknowledged the benefits of using realist review in intervention development [90], but such methods have not previously been used in hip fracture rehabilitation research. This paper adds to the understanding of how a realist review can be used in conjunction with other methods to develop complex interventions which link individual intervention components with underlying programme theories.

The findings from the individual work packages are supported by existing literature. A qualitative study of physiotherapists' perceptions of rehabilitation also showed that tailoring of care to patient's individual needs, based on their own goals and level of support available, was an important component of successful rehabilitation [91]. A previous qualitative study exploring mobility levels pre- and post-fracture also reported that fear of falling, lack of confidence and reliance on others had an impact on patients' experiences of rehabilitation [92]. This study highlighted the need to include psychological components in rehabilitation interventions, supporting our findings about the importance of improving self-efficacy and confidence in hip fracture patients. Our finding concerning patients' need for information from healthcare professionals and its importance in successful rehabilitation has also been previously identified [93]. A study into the challenges of team working in the rehabilitation of hip fracture patients found that there were breakdowns in communication within multidisciplinary teams and issues relating to the organisation of resources and

For peer review only - http://bmjopen.bmj.com/site/about/guidelines.xhtml

For peer review only

services, which led to variation in patient care [94]. Our intervention aims to address this by co-ordinating care through the means of a patient-held goal-setting diary.

This was the first realist review of rehabilitation following hip fracture and the first UK wide survey aiming to describe rehabilitation for patients following hip fracture across acute and community settings since the introduction of NICE recommendations for rehabilitation in 2011 [5]. As a realist review rather than a systematic review was performed we did not attempt to summarise all of the evidence and judge whether rehabilitation programmes were effective, but rather sought to build an explanatory account of mechanisms behind rehabilitation. Whilst a good range of respondents were sampled in the survey, it was not possible to sample settings, therapists and community service managers proportionately, which may impact on how representative findings are of the whole UK. Similarly, focus groups findings relate specifically to the location we recruited from as this was the proposed setting for the delivery of the enhanced rehabilitation programme. We had also hoped to purposively sample patients with different levels of disability who had received different types of rehabilitation, however it was not possible to identify these criteria from electronic medical records. Participants had a range of ages and experiences across the groups, though we were unable to sample those who were living independently prior to hip fracture, but who now lived in residential or nursing care, and those with cognitive impairment.

Implications for future research and practice

Important implications for practice are the routine assessment of psychological variables and the inclusion of psychological components in rehabilitation interventions. This study demonstrated the potential benefits of using a realist approach to complex intervention development and how a realist review could be used in conjunction with other established

For peer review only - http://bmjopen.bmj.com/site/about/guidelines.xhtml

methods to provide an evidence base for a hip fracture rehabilitation intervention. This approach may be beneficial for developing complex interventions in other clinical areas and can be used to provide theories of how specific intervention components will facilitate their intended outcomes. The next phase in the MRC framework for evaluating complex interventions [12] was to test the feasibility of methods for a future trial of the developed intervention by testing its acceptability in a phase II feasibility study [89, 95].

Review registration

Williams N, Din N, Rycroft-Malone J, Wilkinson C, Edwards R, Charles J. Fracture in the Elderly Multidisciplinary Rehabilitation (FEMuR): developing a multidisciplinary rehabilitation package following hip fracture. PROSPERO 2012:CRD42012003208. Available from http://www.crd.york.ac.uk/PROSPERO/display_record.asp?ID=CRD42012003208. 2012.

Declarations

Ethics, consent and permissions

Ethical approval was granted for the study by UK NHS North Wales Research Ethics Committee – West. Ref 12/WA/0355. All participants gave informed consent to participate in accordance with the procedures approved by the committee.

Consent for publication

Individual data has been anonymised, and consent was obtained for use in publication.

Availability of data

For peer review only - http://bmjopen.bmj.com/site/about/guidelines.xhtml

The datasets generated and analysed during the current study are available from the corresponding author on reasonable request.

Conflict of interest statement

None declared.

Funding

This research was funded by the Health Technology Assessment (HTA) programme of the National Institute for Health Research (NIHR). (HTA reference 11/33/03). The views and opinions expressed therein are those of the authors and do not necessarily reflect those of the HTA programme, NIHR, the National Health Service, or the Department of Health.

Author contributions

NHW – CI responsible for study design, conduct and analysis, led intervention development, led writing of manuscript and is guarantor; JLR – conducted focus group analysis and contributed to survey analysis, led writing of manuscript, development of intervention materials; NUD – conducted realist review, contributed to writing manuscript; MW – conducted survey analysis, contributed to writing of manuscript; CH – trial management, input to study and survey design, oversight of intervention development, conducted focus groups and survey, initial focus group analysis; JC – assisted with realist review; ZH – input to study design, design of survey and initial survey analysis; VM – Co-I responsible for study design, provided health psychology expertise and methodological oversight; SA – Co-I, consultant orthogeriatrician, provided orthogeriatric expertise and input on intervention design; AL – Co-I responsible for study design, provided methodological oversight; CS – Co-I contributing to methodology and study design, provided physiotherapy and rehabilitation

For peer review only - http://bmjopen.bmj.com/site/about/guidelines.xhtml

For peer review only

expertise and input on intervention design; PL - Co-I contributing to methodology and study design, provided physiotherapy and rehabilitation expertise and input on intervention design; CW – Co-I contributing to study design and methodology; JRM – Co-I providing realist review expertise and methodological input. All authors were involved in writing and reviewing of the manuscript and decisions on final content.

Acknowledgements

We would like to thank the patients, carers and staff who took part in the focus groups and survey, especially Glenys Mutter who maintains the Hip Fracture Database at Ysbyty Glan Clwyd and assisted us with identifying potential focus group participants.

We would also like to thank:

Dr Julia Hiscock for assistance with focus groups and consultation on qualitative analysis; Aaron Pritchard for consultation on qualitative analysis; Barbara France for running the electronic literature searches and study document retrieval; Annie Hendry for help in extracting the data from the included studies; Dr Richa Sinha for helping during reference screening and conceptual categorisation of potentially relevant studies for the realist review; Patricia Masterson Allgar and Professor Christopher Burton who were participants in the realist review workshops; John Spalding/ Bangor University library staff for helping in electronically inaccessible document retrieval through inter library loans.

For peer review only

References

1. Johansen A, et al., *National Hip Fracture Database. National report 2013*, C.E.a.E. Unit, Editor 2013, Royal College of Physicians: London.
2. National Osteoporosis Foundation *Osteoporosis: review of the evidence for prevention, diagnosis and treatment and cost-effectiveness analysis. Executive summary.* Osteoporosis International, 1998. **4**(S3-6).
3. Schnell, S., et al., *The 1-year mortality of patients treated in a hip fracture program for elders.* Geriatr Orthop Surg Rehabil, 2010. **1**(1): p. 6-14.
4. Roche, J.J., et al., *Effect of comorbidities and postoperative complications on mortality after hip fracture in elderly people: prospective observational cohort study.* BMJ, 2005. **331**(7529): p. 1374.
5. Bertram, M., et al., *Review of the long-term disability associated with hip fractures.* Injury Prevention, 2011. **17**(6): p. 365-370.
6. Harvey, N., E. Dennison, and C. Cooper, *Osteoporosis: impact on health and economics.* Nat Rev Rheumatol, 2010. **6**(2): p. 99-105.
7. *National Osteoporosis Society. Key facts and figures. Accessed September 2014. Available at http://www.nos.org.uk/page.aspx?pid=328*.
8. National Clinical Guideline Centre. *Hip fracture: the management of hip fracture in adults | Guidance and guidelines | NICE. National Institute of Health and Care Excellence 2011. Accessed 2014. Available at http://www.nice.org.uk/guidance/cg124*.
9. Handoll, H.H., et al., *Multidisciplinary rehabilitation for older people with hip fractures.* Cochrane Database of Systematic Reviews, 2009(4).
10. Handoll, H.H., C. Sherrington, and J.C. Mak, *Interventions for improving mobility after hip fracture surgery in adults.* Cochrane Database of Systematic Reviews, 2011(3).
11. Crotty, M., et al., *Rehabilitation interventions for improving physical and psychosocial functioning after hip fracture in older people.* Cochrane Database of Systematic Reviews, 2010(1).
12. Medical Research Council, *Developing and evaluating complex interventions: new guidance*, 2008.
13. Wong, G., et al. *Realist Synthesis: RAMESES Training Materials*. 2013; Available from: http://www.ramesesproject.org/media/Realist_reviews_training_materials.pdf.
14. Wong, G., et al., *Development of methodological guidance, publication standards and training materials for realist and meta-narrative reviews: the RAMESES (Realist And Meta-narrative Evidence Syntheses – Evolving Standards) project.*, H.S.a.D. Research., Editor 2014, NIHR Journals Library.: Southampton (UK).
15. Pawson, R., et al., *Realist review - a new method of systematic review designed for complex policy interventions.* J Health Serv Res Policy, 2005. **10**.
16. Wong, G., et al., *Realist methods in medical education research: what are they and what can they contribute?* Med Educ, 2012. **46**.
17. Rycroft-Malone, J., et al., *Realist synthesis: illustrating the method for implementation research.* Implement Sci, 2012. **7**.
18. Williams, N.H., et al., *Developing a multidisciplinary rehabilitation package following hip fracture and testing in a randomised feasibility study: Fracture in the Elderly Multidisciplinary Rehabilitation (FEMuR).* Health Technology Assessment, 2017. **21**.
19. Lafferty, G., *Community-based alternatives to hospital rehabilitation services: a review of the evidence and suggestions for approaching future evaluations.* Reviews in Clinical Gerontology, 1996. **6**(02): p. 183-194.
20. Handoll, H.H.G. and C. Sherrington, *Mobilisation strategies after hip fracture surgery in adults.* Cochrane Database of Systematic Reviews, 2007(1): p. CD001704.

For peer review only - http://bmjopen.bmj.com/site/about/guidelines.xhtml

21. Ward, D., et al., *Care home versus hospital and own home environments for rehabilitation of older people.* Cochrane Database of Systematic Reviews,, 2008. **DOI: 10.1002/14651858.CD003164.pub2.**
22. Cameron, I.D., et al., *Co-ordinated multidisciplinary approaches for inpatient rehabilitation of older patients with proximal femoral fractures.* Cochrane Database of Systematic Reviews, 2001(3): p. CD000106.
23. Auais, M., O. Eilayyan, and N.E. Mayo, *Extended Exercise Rehabilitation After Hip Fracture Improves Patients' Physical Function: A Systematic Review and Meta-Analysis.* Physical Therapy, 2012.
24. Beaupre, L.A., et al., *Best Practices for Elderly Hip Fracture Patients.* Journal of General Internal Medicine, 2005. **20**(11): p. 1019-1025.
25. Cameron, I.D., *Coordinated multidisciplinary rehabilitation after hip fracture.* Disability & Rehabilitation, 2005. **27**(18/19): p. 1081-1090.
26. Chudyk, A.M., et al., *Systematic review of hip fracture rehabilitation practices in the elderly.* Archives of Physical Medicine & Rehabilitation, 2009. **90**(2): p. 246-262.
27. Feehan, L.M., et al., *Exercise prescription after fragility fracture in older adults: a scoping review.* Osteoporosis International, 2011. **22**(5): p. 1289-322.
28. Halbert, J., et al., *Multi-disciplinary rehabilitation after hip fracture is associated with improved outcome: a systematic review.* Journal of Rehabilitation Medicine (Stiftelsen Rehabiliteringsinformation), 2007. **39**(7): p. 507-512.
29. Leigheb, F., et al., *The Effect of Care Pathways for Hip Fractures: A Systematic Review.* Calcified Tissue International, 2012. **91**(1): p. 1-14.
30. Mehta, S.P. and J.-S. Roy, *Systematic review of home physiotherapy after hip fracture surgery.* Journal of Rehabilitation Medicine (Stiftelsen Rehabiliteringsinformation), 2011. **43**(6): p. 477-480.
31. Moffa-Trotter, M.E. and W.K. Anemaet, *Cost Effectiveness of Home Rehabilitation: A Literature Review.* Topics in Geriatric Rehabilitation, 1999. **14**(4): p. 1-33.
32. Nigwekar, S.U., J. Rajda, and S.D. Navaneethan, *Hospitalist care and length of stay in patients with hip fracture: A systematic review.* Archives of Internal Medicine, 2008. **168**(9): p. 1010-1011.
33. Sherrington, C., *The effects of exercise on physical ability following fall-related hip fracture.* University of New South Wales (Australia), 2001: p. p. pages unknown.
34. Smith, T.O., et al., *Early rehabilitation following less invasive surgical stabilisation plate fixation for distal femoral fractures.* Physiotherapy, 2009. **95**(2): p. 61-75.
35. Toussant, E.M. and M. Kohia, *A Critical Review of Literature Regarding the Effectiveness of Physical Therapy Management of Hip Fracture in Elderly Persons.* The Journals of Gerontology Series A: Biological Sciences and Medical Sciences, 2005. **60**(10): p. 1285-1291.
36. Yamamoto, N., *Rehabilitation and mobilization in hip fracture patients.* Clinical Calcium, 2010. **20**(9): p. 1402-6.
37. Allen, J., et al., *Rehabilitation in Patients with Dementia Following Hip Fracture: A Systematic Review.* Physiotherapy Canada, 2012. **64**(2): p. 190-201.
38. Bachmann, S., et al., *Inpatient rehabilitation specifically designed for geriatric patients: systematic review and meta-analysis of randomised controlled trials.* BMJ, 2010. **340**: p. c1718.
39. Cameron, I., et al., *Geriatric rehabilitation following fractures in older people: a systematic review.* Health Technology Assessment, 2000. **4**(2): p. 105.
40. Cameron, I.D., *Models of rehabilitation -- commonalities of interventions that work and of those that do not.* Disability & Rehabilitation, 2010. **32**(12): p. 1051-1058.
41. Chong, C.P., J. Savige, and W.K. Lim, *Orthopaedic-geriatric models of care and their effectiveness.* Australasian Journal on Ageing, 2009. **28**(4): p. 171-176.

For peer review only - http://bmjopen.bmj.com/site/about/guidelines.xhtml

42. Drenth, J.C. and H. J.S.M., *Assessment of physical fitness using upper extremity tests in elderly patients during the non-weight bearing stage after hip fracture: a systematic review* [Dutch]. Nederlands Tijdschrift Voor Fysiotherapie, 2012. **122**(3): p. 104-110.
43. Egan M, J.S., Byrne K, Wells J, Stolee P, *Factors associated with a second hip fracture: a systematic review.* Clinical Rehabilitation 2008. **22**: p. 272-282.
44. Egol, K.A., K.J. Koval, and J.D. Zuckerman, *Functional Recovery Following Hip Fracture in the Elderly.* Journal of Orthopaedic Trauma, 1997. **11**(8): p. 594-599.
45. Green, J., et al., *Burden and medical needs in older patients with hip fractures and muscle atrophy or weakness.* Osteoporosis International, 2012. **23**: p. S93-S94.
46. Hu, F., et al., *Preoperative predictors for mortality following hip fracture surgery: A systematic review and meta-analysis.* Injury, 2012. **43**(6): p. 676-685.
47. Jelicic, M., G.I.J. Kempen, and L.M. van Eijk, *Do psychosocial factors affect recovery from hip fracture in the elderly? A review of the literature.* Journal of Rehabilitation Sciences, 1996. **9**(3): p. 77-81.
48. Mauffrey, C., *The management of subcapital fractures in the elderly population.* European Journal of Orthopaedic Surgery & Traumatology, 2009. **20**(5): p. 359-364.
49. Muir, S.W. and A.M. Yohannes, *The Impact of Cognitive Impairment on Rehabilitation Outcomes in Elderly Patients Admitted with a Femoral Neck Fracture: A Systematic Review.* Journal of Geriatric Physical Therapy, 2009. **32**(1): p. 24-32.
50. Robinson, K.M., *Efficacy of home care rehabilitation interventions.* Annals of Long-Term Care, 2000. **8**(9): p. 68-71.
51. Romeo, R., et al., *Treatment and prevention of depression after surgery for hip fracture in older people: cost-effectiveness analysis.* Journal of Affective Disorders, 2011. **128**(3): p. 211-9.
52. Visschedijk, J., et al., *Fear of falling after hip fracture: a systematic review of measurement instruments, prevalence, interventions, and related factors.* Journal of the American Geriatrics Society, 2010. **58**(9): p. 1739-1748.
53. Wang, X. and L.J. Emery, *Cognitive status after hip replacement.* Physical & Occupational Therapy in Geriatrics, 2002. **21**(1): p. 51-64.
54. *Scottish Intercollegiate Guidelines Network (SIGN) Management of hip fracture in older people: a national clinical guideline.* Edinburgh, SIGN, 2010.
55. Hip Fractures. October 2014. Agency for Healthcare Research and Quality, R., MD. http://www.ahrq.gov/research/findings/evidence-based-reports/hipfractp.html.
56. *Health Quality Ontario; Ministry of Health and Long-Term Care. Quality-Based Procedures: Clinical Handbook for Hip Fracture. Toronto, ON: Health Quality Ontario; 2013 May. 97 p. Available from: http://www.hqontario.ca/evidence/publications-and-ohtac-rec.*
57. *Minimum Standards for the Management of Hip Fracture in the Older Person. Agency for Clinical Innovation, New South Wales, Austrailia. Available at http://www.aci.health.nsw.gov.au/__data/assets/pdf_file/0004/222727/Minimum-Standards-for-the-Management-of-Hip-Fractures.pdf.*
58. *New Zealand Guidelines Group (2003). Acute management and immediate rehabilitation after hip fracture amongst people ages 65 years and over. Wellington, New Zealand. Available at http://www.moh.govt.nz/NoteBook/nbbooks.nsf/0/BF485CCA409F38C5CC256DCE0070F7B5/$file/Hip_Fracture_Management.pdf.*
59. Resnick, B., et al., *Adherence to an Exercise Intervention Among Older Women Post Hip Fracture.* Journal of clinical sport psychology, 2008. **2**(1): p. 41-56.
60. Resnick, B. and M.P. Daly, *The effect of cognitive status on outcomes following rehabilitation.* Family medicine, 1997. **29**(6): p. 400-405.
61. Resnick, B., et al., *Evaluating the components of the Exercise Plus Program: rationale, theory and implementation.* Health Education Research, 2002. **17**(5): p. 648-658.

For peer review only - http://bmjopen.bmj.com/site/about/guidelines.xhtml

62. Resnick, B., et al., *The Relationship Between Psychosocial State and Exercise Behavior of Older Women 2 Months After Hip Fracture.* Rehabilitation Nursing, 2007. **32**(4): p. 139-149.
63. Resnick, B., et al., *The Exercise Plus Program for Older Women Post Hip Fracture: Participant Perspectives.* The Gerontologist, 2005. **45**(4): p. 539-544.
64. Resnick, B., et al., *Testing the effectiveness of the exercise plus program in older women post-hip fracture.* Annals of Behavioral Medicine, 2007. **34**(1): p. 67-76.
65. Pearson M, H.H., Cooper C, Shepperd S, Pawson R, Anderson R, *Intermediate care: a realist review and conceptual framework.*, in *NIHR Service Delivery and Organisation Programme*2013.
66. Ritzer, G., *Meta-theorizing in Sociology.*1991, Lexington, MA: Lexington Books.
67. Roen, K., et al., *Extending systematic reviews to include evidence on implementation: Methodological work on a review of community-based initiatives to prevent injuries.* Social Science & Medicine, 2006. **63**(4): p. 1060-1071.
68. Pluye P, Robert E, and e.a. Cargo M, *Proposal: A mixed methods appraisal tool for systematic mixed studies reviews.* 2011.
69. Ritchie, J.S., L, *Qualitative data analysis for applied policy research.*, in *Analysing Qualitative Data* B.A.B. R.G, Editor 1994, Routledge: London. p. 173–194.
70. Bellelli, G., et al., *Action Observation Treatment Improves Recovery of Postsurgical Orthopedic Patients: Evidence for a Top-Down Effect?* Archives of Physical Medicine & Rehabilitation, 2010. **91**(10): p. 1489-1494.
71. Saltvedt, I., et al., *Development and delivery of patient treatment in the Trondheim Hip Fracture Trial. A new geriatric in-hospital pathway for elderly patients with hip fracture.* BMC Research Notes, 2012. **5**: p. 355.
72. Rolland, Y., et al., *Rehabilitation outcome of elderly patients with hip fracture and cognitive impairment.* Disability & Rehabilitation, 2004. **26**(7): p. 425-31.
73. Stenvall, M., et al., *Improved performance in activities of daily living and mobility after a multidisciplinary postoperative rehabilitation in older people with femoral neck fracture: a randomized controlled trial with 1-year follow-up.* Journal of Rehabilitation Medicine (Stiftelsen Rehabiliteringsinformation), 2007. **39**(3): p. 232-238.
74. Young, Y. and B. Resnick, *Don't worry, be positive: improving functional recovery 1 year after hip fracture.* Rehabilitation Nursing, 2009. **34**(3): p. 110-117.
75. Jackson, J. and J. Schkade, *Occupational adaptation model versus biomechanical-rehabilitation model in the treatment of patients with hip fractures.* American Journal of Occupational Therapy, 2001. **55**(5): p. 531-7.
76. Godfrey, M. and J. Townsend, *Older People in Transition From Illness to Health: Trajectories of Recovery.* Qualitative Health Research, 2008. **18**(7): p. 939-951.
77. Manthorpe, J. and M. Cornes, *Intermediate Care: Older People's Involvement and Experiences.* Journal of Integrated Care, 2004. **12**(6): p. 43-48.
78. Crotty, M., et al., *Early discharge and home rehabilitation after hip fracture achieves functional improvements: a randomized controlled trial.* Clinical Rehabilitation, 2002. **16**(4): p. 406-13.
79. Sirkka, M. and I. Bränholm, *Consequences of a hip fracture in activity performance and life satisfaction in an elderly Swedish clientele.* Scandinavian Journal of Occupational Therapy, 2003. **10**(1): p. 34-39.
80. Ziden, L., C. Wenestam, and M. Hansson-Scherman, *A life-breaking event: early experiences of the consequences of a hip fracture for elderly people.* Clinical Rehabilitation, 2008. **22**(9): p. 801-11.
81. Bauerle, D., N. Specht-Leible, and E. Voss, *Hip fracture - Changes in need of help and care - Cluster analysis - Formal and informal support. [German] Veranderungen des hilfe- Und pflegebedarfs nach huftnahen frakturen im hoheren lebensalter.* Zeitschrift fur Gerontologie und Geriatrie, 2004. **37**(5): p. 351-353.

For peer review only

For peer review only - http://bmjopen.bmj.com/site/about/guidelines.xhtml

82. Huang, T. and G. Acton, *Ways to maintain independence among Taiwanese elderly adults with hip fractures: a qualitative study.* Geriatric Nursing, 2009. **30**(1): p. 28-35.
83. Lieberman, D. and M. Friger, *Inpatient rehabilitation outcome after hip fracture surgery in elderly patients: a prospective cohort study of 956 patients.* Archives of Physical Medicine & Rehabilitation, 2006. **87**(2): p. 167-171.
84. McMillan, L., et al., *A grounded theory of taking control after fall-induced hip fracture.* Disability & Rehabilitation, 2012. **34**(26): p. 2234-2241.
85. Resnick, B., et al., *Factors that influence exercise activity among women post hip fracture participating in the Exercise Plus Program.* Clin Interv Aging, 2007. **2**(3): p. 413-27.
86. Bandura, A., *Self-efficacy: The exercise of control*1997, New York, NY, US: W H Freeman/Times Books/ Henry Holt & Co. ix, 604.
87. Morrison, V.L., et al., *Improving Emotional Outcomes following Acute Stroke: A Preliminary Evaluation of a Work-Book Based Intervention.* Scottish Medical Journal, 1998. **43**(2): p. 52-53.
88. Johnston, M., et al., *Recovery from disability after stroke as a target for a behavioural intervention: Results of a randomized controlled trial.* Disability and Rehabilitation, 2007. **29**(14): p. 1117-1127.
89. Williams, N.H., et al., *Fracture in the Elderly Multidisciplinary Rehabilitation (FEMuR): study protocol for a phase II randomised feasibility study of a multidisciplinary rehabilitation package following hip fracture [ISRCTN22464643].* Pilot and Feasibility Studies, 2015. **1**(1): p. 1-22.
90. Pearson, M., et al., *Using realist review to inform intervention development: methodological illustration and conceptual platform for collaborative care in offender mental health.* Implement Sci, 2015. **10**: p. 134.
91. Taylor, N.F., Harding, K.E., Dowling, J., Harrison, G, *Discharge planning for patients receiving rehabilitation after hip fracture: A qualitative analysis of physiotherapists' perceptions.* Disability and Rehabilitation, 2010. **32**(6): p. 492-499.
92. Taylor, N.F., Barelli, C and Harding, K.E, *Community ambulation before and after hip fracture: a qualitative analysis.* Disability and Rehabilitation, 2010. **32**(15): p. 1281-90.
93. McMillan, L., et al., *'Balancing risk' after fall-induced hip fracture: the older person's need for information.* International Journal of Older People Nursing, 2014. **9**(4): p. 249-257.
94. Long, A.F., R. Kneafsey, and J. Ryan, *Rehabilitation practice: challenges to effective team working.* International Journal of Nursing Studies, 2003. **40**(6): p. 663-673.
95. Williams, N.H., et al., *Fracture in the Elderly Multidisciplinary Rehabilitation (FEMuR): a phase II randomised feasibility study of a multidisciplinary rehabilitation package following hip fracture.* BMJ Open, 2016. **6**(10): p. e012422.

Fig 1. Development of programme theories for informing the content of the enhanced rehabilitation intervention.

Fig. 2. Frequency of rehabilitation visits following hip fracture

Fig. 3. Proposed outcomes of individual intervention components target specific programme theories.

For peer review only - http://bmjopen.bmj.com/site/about/guidelines.xhtml

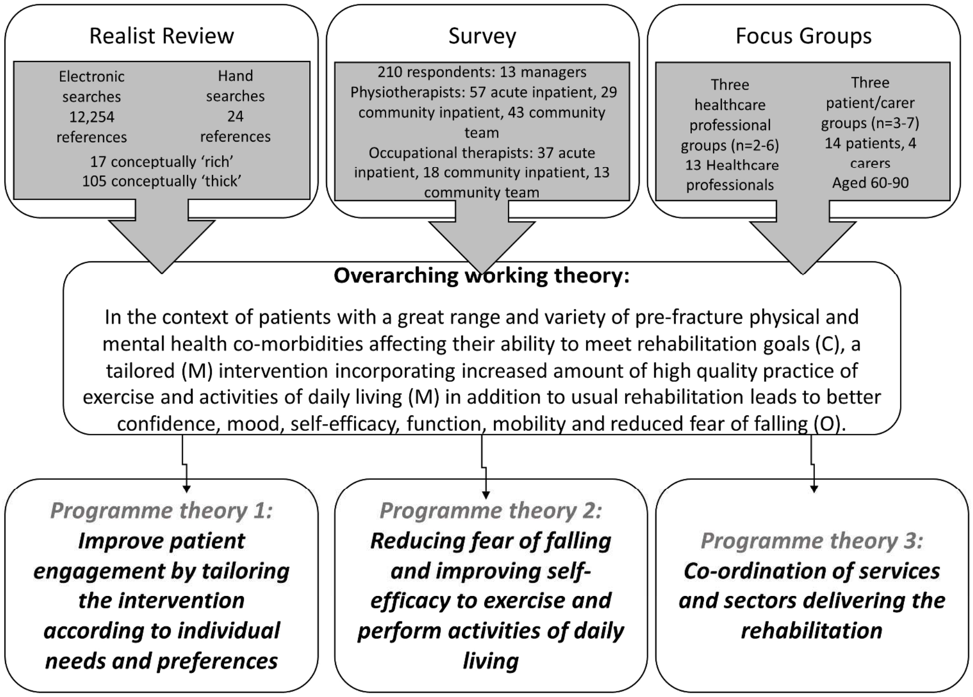

Fig 1. Development of programme theories for informing the content of the enhanced rehabilitation intervention.

254x190mm (300 x 300 DPI)

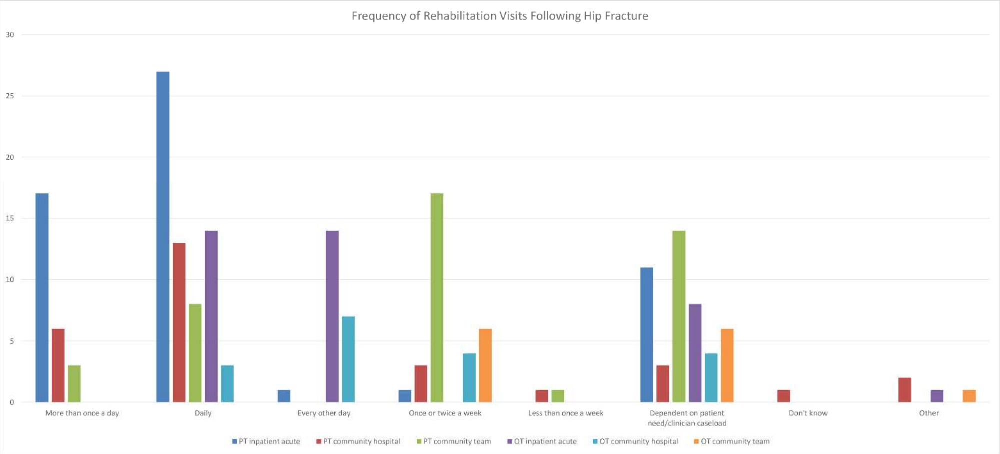

Fig. 2. Frequency of rehabilitation visits following hip fracture

345x157mm (300 x 300 DPI)

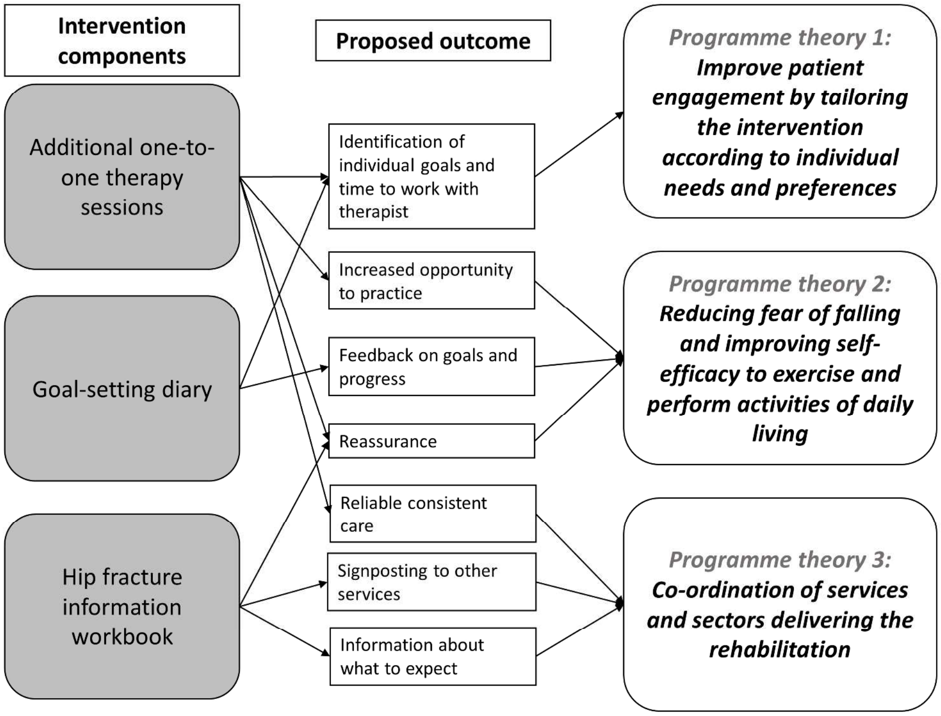

Fig. 3. Proposed outcomes of individual intervention components target specific programme theories.

254x190mm (300 x 300 DPI)

For peer review only - http://bmjopen.bmj.com/site/about/guidelines.xhtml

Appendix 1 – List of databases searched for realist review.

- MEDLINE
- MEDLINE In-Process & Other Non-Indexed Citations
- OLDMEDLINE
- EMBASE
- Cumulative Index to Nursing and Allied Health Literature (CINAHL)
- Allied and Complimentary Medicine Database (AMED)
- British Nursing Index
- Health Management Information Consortium (HMIC)
- PsychINFO
- Cochrane Central Register of Controlled Trials (CENTRAL)
- Database of Abstracts of Reviews of Effects (DARE)
- Cochrane Database of Systematic Reviews (CDSR)
- Health Technology Assessment (HTA) Database
- NHS Economic Evaluation Database (NHS EED)
- Science Citation Index
- Social Science Citation Index (SSCI)
- Index to Scientific & Technical Proceedings (ISTP)
- Physiotherapy Evidence Database (PEDro)
- BIOSIS
- System for Information on Grey Literature In Europe (SIGLE)
- Web of Knowledge Index of Theses and Dissertations

For peer review only
